# Development of land use regression models for nitrogen dioxide, ultrafine particles, lung deposited surface area, and four other markers of particulate matter pollution in the Swiss SAPALDIA regions

**DOI:** 10.1186/s12940-016-0137-9

**Published:** 2016-04-18

**Authors:** Marloes Eeftens, Reto Meier, Christian Schindler, Inmaculada Aguilera, Harish Phuleria, Alex Ineichen, Mark Davey, Regina Ducret-Stich, Dirk Keidel, Nicole Probst-Hensch, Nino Künzli, Ming-Yi Tsai

**Affiliations:** Department of Epidemiology and Public Health, Swiss Tropical & Public Health Institute, Socinstrasse 57, P.O. Box 4002, Basel, Switzerland; University of Basel, Basel, Switzerland; CESE, Indian Institute of Technology Bombay, Mumbai, India; Department of Environmental & Occupational Health Sciences, University of Washington, Seattle, USA

**Keywords:** SAPALDIA, Air pollution, Long term, Traffic, Particulate matter, Nanoparticles, Land use regression, LUR, NO_2_, PM_2.5_, Absorbance, PM_10_, Coarse fraction, PNC, LDSA

## Abstract

**Background:**

Land Use Regression (LUR) is a popular method to explain and predict spatial contrasts in air pollution concentrations, but LUR models for ultrafine particles, such as particle number concentration (PNC) are especially scarce. Moreover, no models have been previously presented for the lung deposited surface area (LDSA) of ultrafine particles. The additional value of ultrafine particle metrics has not been well investigated due to lack of exposure measurements and models.

**Methods:**

Air pollution measurements were performed in 2011 and 2012 in the eight areas of the Swiss SAPALDIA study at up to 40 sites per area for NO_2_ and at 20 sites in four areas for markers of particulate air pollution. We developed multi-area LUR models for biannual average concentrations of PM_2.5_, PM_2.5_ absorbance, PM_10_, PM_coarse_, PNC and LDSA, as well as alpine, non-alpine and study area specific models for NO_2_, using predictor variables which were available at a national level. Models were validated using leave-one-out cross-validation, as well as independent external validation with routine monitoring data.

**Results:**

Model explained variance (R^2^) was moderate for the various PM mass fractions PM_2.5_ (0.57), PM_10_ (0.63) and PM_coarse_ (0.45), and was high for PM_2.5_ absorbance (0.81), PNC (0.87) and LDSA (0.91). Study-area specific LUR models for NO_2_ (R^2^ range 0.52–0.89) outperformed combined-area alpine (*R*^*2*^ = 0.53) and non-alpine (*R*^*2*^ = 0.65) models in terms of both cross-validation and independent external validation, and were better able to account for between-area variability. Predictor variables related to traffic and national dispersion model estimates were important predictors.

**Conclusions:**

LUR models for all pollutants captured spatial variability of long-term average concentrations, performed adequately in validation, and could be successfully applied to the SAPALDIA cohort. Dispersion model predictions or area indicators served well to capture the between area variance. For NO_2_, applying study-area specific models was preferable over applying combined-area alpine/non-alpine models. Correlations between pollutants were higher in the model predictions than in the measurements, so it will remain challenging to disentangle their health effects.

**Electronic supplementary material:**

The online version of this article (doi:10.1186/s12940-016-0137-9) contains supplementary material, which is available to authorized users.

## Background

Several studies have documented large spatial contrasts in air pollution in European and US cities [1–4]. Land Use Regression (LUR) modeling has become a popular method for explaining the observed contrasts [5–10], as well as estimating outdoor pollution concentrations at the homes of participants of large epidemiological studies [11–14]. LUR relies on a spatially dense air pollution monitoring network, of which each site is characterized by a set of potential “predictor variables”, which are generally derived from Geographic Information Systems (GIS) [7]. In LUR, a regression model is developed which links the air pollution concentrations observed in the network to the most predictive environmental characteristics, such as traffic, land use and population. Depending on the pollutant, LUR modeling has been able to explain a moderate to large amount of spatial variability in concentration for a growing arsenal of pollutants.

Where traditionally, mostly nitrogen dioxides and particulate matter were modeled [7], a few recent LUR studies have also modeled ultrafine particles [15–21], mostly based on mobile [17, 20] or short period monitoring [16, 18, 19, 21], and therefore modeling concentrations which are not necessarily representative for the longer-term average. Long-term, spatially resolved monitoring campaigns for ultrafine particles (UFP) have been uncommon because the condensation particle counters which are typically used for measurements are costly and require daily maintenance. The MiniDisc devices used in our study could be deployed for longer periods with relatively little maintenance.

Typically, LUR models are developed for single cities, metropolitan areas or regions, and applied within the same geographical parameters. Some attempts have been presented to combine study areas [6, 10, 22–24], or transfer LUR models between areas [24–26]. Most studies conclude that locally developed models are favorable over combined-area or transferred models [10, 24–26]. However, it has been mentioned that combined-area models may allow epidemiological studies to pool epidemiological data from different areas and better exploit the between area contrasts, which would substantially increase the exposure range for some pollutants [22]. The background concentration difference between areas is often mentioned as a source of over- or under-prediction when applying these combined-area models, and has been addressed by including indicator variables [6], area-specific regional background [22] or recently satellite-observed background NO_2_ [23], or (like in this paper) larger scale dispersion models [10, 27]. Data for the latter two approaches may not be available for all pollutants, but these approaches have the advantage of being able to interpolate between study areas. The availability of common-source predictor data is crucial to the success of transferring LUR models or combining study areas [22, 25, 26]. If the allocation of land use categories differs between areas, or traffic intensity dataare obtained from different traffic models, this may compromise the quality of a combined-area model [10] or the ability to transfer models between areas [25].

This paper describes the development, performance and validation of multi-area LUR models for nitrogen dioxide (NO_2_), particulate matter <2.5 μm (PM_2.5_), PM_2.5_ filter absorbance, a marker for diesel exhaust particles (PM_2.5_ absorbance), particulate matter <10 μm (PM_10_), the coarse fraction calculated as the difference between PM_10_ and PM_2.5_ (PM_coarse_), particle number concentration (PNC) and the lung deposited surface area (LDSA) of particles. For NO_2_, measurements and models cover all eight areas of the Swiss Study on Air Pollution and Lung and heart Diseases (SAPALDIA). For particle-related markers of air pollution, the study covers four of the eight areas. This paper presents one of the first LUR models representing long-term exposure to PNC and the first model for LDSA of ultrafine particles. Several toxicological studies suggest that the strength of the induced inflammatory response is related to the contact surface area of the pollutant with the alveolar cells, rather than its mass [28, 29], making it a biologically promising metric of exposure [30]. Moreover, the small size of ultrafine particles facilitates translocation into the bloodstream and uptake into cells [31]. However, due to the lack of exposure data, no epidemiological study on long-term effects of air pollution has evaluated the added value of its use. Furthermore, we evaluate the performance of study area-specific and multi-area NO_2_ models to predict concentrations of an independent validation dataset. The LUR models presented in this paper will be applied to the SAPALDIA study population to estimate subjects’ exposure to the above pollutants. The SAPALDIA cohort was initiated in 1991 and had follow-ups in 2002 and 2011 (SAPALDIA 3) [32, 33].

## Methods

We developed LUR models for biannual average concentrations of NO_2_ for eight areas in Switzerland, and for PM_2.5_, PM_2.5_ absorbance, PM_10_, PM_coarse_, PNC and LDSA for four areas, due to financial constraints, using a range of geographic predictor variables. NO_2_ models were developed for all eight areas combined, for each area separately, and for the alpine and non-alpine regions separately. All predictors were available nationwide. We used a supervised stepwise method to develop LUR models, maximizing model explained variance, while allowing only “plausible” directions of effect (e.g. increase in concentration for traffic, decrease in concentration for proximity to green space).

### Air pollution measurement data

The SAPALDIA 3 measurement campaign and results have been described previously [34]. Briefly, eight study areas (Aarau, Basel, Davos, Geneva, Lugano, Montana, Payerne and Wald) were selected to cover the spatial distribution of SAPALDIA 3 cohort addresses (Additional file 1). Forty NO_2_ sampling sites were selected in each study area. In Basel, Geneva, Lugano and Wald, PM_2.5,_ PM_10_, PNC and LDSA measurements were co-located with the NO_2_ samplers at 20 of the 40 sites. NO_2_ was measured with passive diffusion samplers (Passam AG, Männedorf, Switzerland), PM_2.5_ and PM_10_ were collected on filters using Harvard Impactors, PM_2.5_ absorbance was measured as reflectance on PM_2.5_ filters using a smoke stain reflectometer, and PNC and LDSA measurements were conducted with the Miniature Diffusion Size Classifier (MiniDiSC) (Fachhochschule Nordwestschweiz, Switzerland) [35]. All measurements were performed between January 2011 and December 2012. In each study area, regional background, urban background and traffic sites were selected. Because a substantial amount of spatial contrast is traffic-related, we chose to over-represent the number of street sites (±50 % of the total), including a wide range of different traffic intensities and street layouts. In Aarau, Davos, Montana and Payerne, all sites were measured simultaneously, three times for 14 days each in the cold, warm and intermediate seasons. In Basel, Geneva, Lugano and Wald, 20 sites (10 PM + NO_2_ and 10 NO_2_-only) were measured simultaneously for 14 days, while the remaining 20 sites were measured during the subsequent 14 days. This was again repeated 3 times in different seasons. A small number of sites for which valid measurements were only available for 1 season, were excluded from the analysis [34], explaining *n* < 40 for NO_2_ or *n* < 20 for PM_2.5_ or PM_10_ in some study areas (Table 2).

For each site, results from the three individual measurements were averaged to represent a bi-annual average over the years 2011 and 2012, by adjusting for the long-term concentration observed at a reference site which was centrally located in each study area. The temporal correction factor for each measurement was calculated as the ratio between the biannual mean and the average reference site measurement during the particular measurement period. Thus, it is assumed that the temporal variation observed at the fixed site monitor reflects the seasonal pattern of the entire area. This procedure has been extensively described before [34].

### GIS predictor data

Sampling site coordinates were determined manually in GIS by investigators who had personally visited the sites, ensuring that the position was accurate relative to roads, and buildings. For each site, we used GIS to calculate 164 geographic predictor variables. To do this, we made use of digital datasets which were available on the European and National (Switzerland) level. A more detailed description of the predictor variables and how they were derived can be found in Table 1.

**Table 1:** Description of evaluated predictor variables

Source data	Variable name(s)^a^	Description	Unit	Direction of effect	Buffer sizes (m)
Building density	BUILDINGS_X	Total area covered by buildings	m^2^	+	25, 50, 75, 100, 150, 200, 250, 300, 500, 1000, 2000, 5000
Population grid	POP_X	Population count	N(umber)	+	100, 150, 200, 250, 300, 500, 1000, 2000, 5000
CORINE Land Cover ^b^	LDRES_X	Low density residential	m^2^	+	100, 150, 200, 250, 300, 500, 1000, 2000, 5000
	HDRES_X	High density residential	m^2^	+	100, 150, 200, 250, 300, 500, 1000, 2000, 5000
	AIRPORT_X	Airport	m^2^	+	100, 150, 200, 250, 300, 500, 1000, 2000, 5000
	INDUSTRY_X	Industry	m^2^	+	100, 150, 200, 250, 300, 500, 1000, 2000, 5000
	NATURAL_X	Natural	m^2^	-	100, 150, 200, 250, 300, 500, 1000, 2000, 5000
	PORT_X	Port	m^2^	+	100, 150, 200, 250, 300, 500, 1000, 2000, 5000
	URBGREEN_X	Urban green	m^2^	-	100, 150, 200, 250, 300, 500, 1000, 2000, 5000
	UGNL_X	Urban green and natural land	m^2^	-	100, 150, 200, 250, 300, 500, 1000, 2000, 5000
	WATER_X	Water	m^2^	+/−	100, 150, 200, 250, 300, 500, 1000
Road network	ROADLENGTH_X	Total lengths of all roads	m	+	25, 50, 75, 100, 150, 200, 250, 300, 500, 1000, 2000, 5000
	MAJROADLENGTH_X ^c^	Total lengths of all major roads	m	+	25, 50, 75, 100, 150, 200, 250, 300, 500, 1000, 2000, 5000
	TRAFLOAD_X	Total traffic load of roads (sum of (traffic intensity * length of each segment))	Veh · day^−1^ · m	+	25, 50, 75, 100, 150, 200, 250, 300, 500, 1000, 2000, 5000
	TRAFMAJORLOAD_X ^c^	Total traffic load of major roads (sum of (traffic intensity * length of each segment))	Veh · day^−1^ · m	+	25, 50, 75, 100, 150, 200, 250, 300, 500, 1000, 2000, 5000
	HEAVYTRAFLOAD_X	Total heavy traffic load of roads (sum of (traffic intensity * length of each segment))	Veh · day^−1^ · m	+	25, 50, 75, 100, 150, 200, 250, 300, 500, 1000, 2000, 5000
	TRAFNEAR, TRAFMAJOR ^c^, HEAVYTRAFNEAR	(Heavy) traffic intensity on the nearest (major) road	Veh · day^−1^	+	N/A
	INTINVDIST, INTINVMAJDIST ^c^, HEAVYINTINVDIST	Traffic on the nearest (major) road * inverse distance	Veh · day^−1^ · m^−1^	+	N/A
	INVDIST, MAJINVDIST ^c^	Inverse distance to the nearest (major) road	m^−1^	+	N/A
DHM Altitude grid	ALT, LOG_ALT, SQRT_ALT	Altitude, log(altitude) and sqrt(altitude)	m	-	N/A
Dispersion model estimates	NO2_2010, PM25_2010, PM10_2010	Pollumap 2010 prediction for NO_2_, PM_2.5_ and PM_10_	μg/m^3^	+	N/A
Area indicator	Area_BS, Area_DA, Area_GE, Area_LU, Area_MO, Area_PA, Area_WA	Dummy variable for presence of a measurement site in the study areas of Aarau, Basel, Davos, Geneva, Lugano, Montana, Payerne, Wald. The reference is Aarau	Not applicable	+/−	N/A

^a^The suffix “X” is replaced by the buffer size in meters to get the full variable name (e.g. BUILDINGS_100 = area covered by buildings in a 100 m buffer); ^b^CORINE classes were defined as previously in the APMOSPHERE [24, 45] and ESCAPE [5, 6, 46] projects; ^c^ Where major road is defined as a road with ≥5000 vehicles/24 h

The following GIS source data were available:

1. Building density

Building footprints were available for the year 2008 from Vector25, the digital landscape model of Switzerland [36]. Data covered the whole country plus several km beyond the borders. We calculated the total area covered by building footprints in various buffers.

2. Population density

Aggregated census population data at a 100×100m grid were available for 2011 from the Bundesamt für Statistik (BfS) http://www.bfs.admin.ch/bfs/portal/de/index/dienstleistungen/geostat/datenbeschreibung/volks-__gebaeude-0.html for Switzerland only. For the border regions, we obtained a 100×100m CORINE (COoRdination of INformation on the Environment) population grid for 2000 for the surrounding countries (http://www.eea.europa.eu/data-and-maps/data/population-density-disaggregated-with-corine-land-cover-2000-2#tab-gisdata). The two grids were combined, adjusting for an average 4.65 % population growth in France, Germany, Italy and Austria over 2000–2011 (Additional file 2).

3. Land use

Digital land cover (CORINE CLC-2006 Version 13, 02–2010) data were available from the European Environment Agency for the year 2006 for all of Europe including Switzerland. We calculated the surface area of 6 land use categories (high density residential, low density residential, airport, industry, natural and port), following re-classifications previously adopted in the ESCAPE [5, 6] and APMOSPHERE [37] projects.

4. Digital road network

A digital road network (Vector 25) with resolution 1:25,000 was available from Swiss Topo (Bundesamt fur Landestopografie) at a national level for the year 2008, with modeled traffic intensity data for the same year. Road class (total 32 classes) and percentage heavy traffic were also available. Roads were distinguished as major if the traffic intensity was ≥ 5000 vehicles/24 h.

5. Altitude

A swiss elevation map with a resolution of 200 m was available from the Bundesamt für Landestopografie (Federal Office for Topography), SwissTOPO, website: http://www.swisstopo.admin.ch/internet/swisstopo/de/home/products/height/dhm25.html. Sampling points were directly overlaid with this height grid.

6. Dispersion model

PolluMap Gaussian dispersion model estimates were available for NO_2_, PM_2.5_ and PM_10_ at a 200×200m resolution, for the year 2010. These dispersion models have been shown to compare well to measurements, and details of their development have been published previously [38]. Sampling points were directly overlaid with the grids.

Few sites were within 500 m of a port or industrial area, or within 25 m of a major road, resulting in many 0-values for the smaller buffers of these land-use classes and traffic variables. Occasionally, these variables are selected as predictor variables, but this causes coefficients to be estimated with a lot of weight given to relatively few sites. This causes high Cook’s distance for the sites in question, and often unstable coefficients, which do not hold up in cross-validation, resulting in large differences between the model R^2^ and LOOCV R^2^, as previously noted for other LUR studies [5, 6]. The same happens for predictor variables which include extreme outliers. Therefore, we a priori restricted the set of eligible predictors, eliminating those which 1) had fewer than five sites with a value other than the most common value (typically 0); 2) had a maximum which was more than 3 times the P10-P90 range above P90, or 3) had a minimum which was more than 3 times the P10-P90 range under P10. The latter 2 criteria are based on the generally accepted criteria for outliers (lower limit P25-3*(P75-P25), upper limit P75 + 3*(P75-P25), but are less restrictive. The elimination process was repeated for each model, and so eligible variables varied, based on the number of sites, and on the selection of sites included in the model.

### LUR model development

Linear regression models were developed using a supervised forward selection procedure, first evaluating univariate regressions. The corrected bi-annual average concentrations were evaluated against all eligible potential predictors. The predictor giving the highest adjusted explained variance (adjusted R^2^) was selected for inclusion in the model if the direction of effect was as defined a priori and the *P*-value was <0.10, following procedures used before [5, 6]. Subsequently, we evaluated if any of the remaining predictor variables further improved the model adjusted R^2^ by at least 0.01. Again, we selected the predictor giving the highest gain in adjusted R^2^, if it had a *P*-value <0.10 and the expected direction of effect. Additional variables were not selected if they changed the direction of effect of one of the previously included variables. This process continued until there were no more variables which fit the criteria, and improved the model adjusted R^2^ by at least 0.01.

As final steps, variables with a *p*-value above 0.10 were removed from the model. Furthermore, we checked that all Variance Inflation Factors (VIF) were lower than 3 (ensuring the absence of collinearity), and that Cook’s Distance values were below 1, ensuring the absence of highly influential observations disproportionately influencing a specific variable’s coefficient.

All analyses were done using SAS version 9.4. The Moran’s I statistic was calculated to indicate spatial autocorrelation of the model residuals (with weights proportional to the inverse distance squared). In addition, the significant dependence of model residuals on the study area was tested for the multi-area models (PROC GLM, with class variable for study area).

The modelling procedure is described in more detail in Additional file 5, Table 1. In the first phase of our modelling effort, we combined all measurement sites where the pollutant in question was measured to fit a single LUR model for each pollutant. We considered, but did not force in PolluMap model NO_2_ (for pollutant NO_2_), Pollumap PM_2.5_ (for pollutants PM_2.5_ and PM_2.5_ absorbance) and PM_10_ (for pollutants PM_10_ and PM_coarse_). This way, we ensured continuity of exposure characterization across areas, and reduced the risk of over-fitting, which is higher for models developed based on a limited number of training sites [39–41]. No dispersion model predictors were evaluated for the main PNC and LDSA models. In the second phase (model 2, Additional file 5, Table 1), we addressed those multi-area models which did not adequately model between-area variability (resulting in significant spatial autocorrelation in the residuals). We tried to force PolluMap NO_2_ estimates into the NO_2_ model, to consider PolluMap NO_2_ for the PM_2.5_ absorbance model, and to consider PM_2.5_ and PM_10_ PolluMap for the PNC and LDSA models, In the third phase, we introduced area-indicators to account for between-area variance. These indicators were introduced to the model as dummy variables for each site in the area of Geneva, Lugano or Wald (Aarau being the reference). Ultimately, in the 4^th^ phase,, we fitted an area-specific NO_2_ model for each study area to optimally capture local contrasts. In addition, we fitted an alpine (study areas Davos and Montana) and a non-alpine model (study areas Aarau, Basel, Geneva, Lugano, Payerne, Wald), retaining the option of making model predictions outside of the eight study areas, for sites above and below 1000 m, respectively. This was only possible for NO_2_, where 40 measurement sites per study area were available. We did not consider building local models for pollutants with only few sites (≤20) available per study area. The models that were ultimately selected for the epidemiological application are shown in the main paper. The selection process and the disregarded alternative models are shown in Additional file 5.

### Validation and quality assurance

Model performance was evaluated in three ways: firstly by leave-one-out cross validation (LOOCV), where each site was sequentially left out from the model while the included variables were left unchanged. Each site’s value was then predicted from the model based on the n-1 sites, after which the R^2^ between observed and predicted values was calculated. Secondly, and only for those models combining multiple study areas, all sites from one particular study area were left out (leave-one-area-out cross validation (LOAOCV), while the remaining sites from the other areas were used to predict their values. The variables in the model were left unchanged. The predicted concentrations from the LOAOCV were compared to the measured concentrations, showing the degree of over- or under-prediction per study area.

Thirdly, external validation was performed using a set of routine monitoring stations from the National air quality monitoring network (NABEL) and several cantonal air monitoring agencies. We gathered data from all available stations in Switzerland which had annual average concentrations available for both 2011 and 2012 for NO_2_ (102 stations), PM_2.5_ (10 stations), elemental carbon (EC) as a marker of PM_2.5_ absorbance (these metrics are known to correlate well from previous studies [3], 18 stations), PM_10_ (82 stations) and PNC (13 stations). PM_coarse_ was calculated as the difference between PM_10_ and PM_2.5_ for 10 stations. No routine monitoring data was available for LDSA, since this is not measured as part of any routine monitoring networks. The predictor variables selected for the models were also extracted for these stations. To avoid predictions outside of the plausible concentration range, predictor values were truncated to the minimum and maximum values present in the various training data sets which were used to develop the LUR models [42]. Predictors derived from dispersion models were not truncated: these were assumed to reflect regional background pollution levels, and were therefore allowed to vary beyond the range present in the training dataset. For PM_2.5_, PM_10_ and PM_coarse_, LUR models were then applied to all available stations. The PM_2.5_ absorbance and PNC models, which included area indicators, were applied to all stations which fell within 20 km of any of the measurement sites used to develop the model for that specific area. This resulted in 5 successful predictions for PM_2.5_ absorbance and 4 for PNC. Reducing the inclusion criterion further (e.g. to 10 km) would have resulted in too few sites (≤3) to allow any comparison. For NO_2_, we applied the area-specific models to stations within 10 km of the sites used for the modelling, the alpine models for stations above 1000 m and the non-alpine model for stations below 1000 m. Model predictions were then compared to the two-year (2011–2012) measured average concentrations, to best represent the time period for which the models were developed.

### Sensitivity analyses

To allow a more direct comparison of model performance between pollutants, we additionally fitted an NO_2_ model for the same four study areas which also contributed to the PM and UFP models (Basel, Geneva, Lugano and Wald). We explored whether applying the local NO_2_ models within 10 km of the study areas, and alpine and non-alpine models everywhere else, gave the most accurate predictions (in comparison to applying alpine and non-alpine models only, or applying local models within 20 km of the study areas).

## Results

Pollutant distribution characteristics are shown in Table 2 and are available in more detail in Eeftens et al. 2015 [34]. For all pollutants, substantial variation was present between as well as within the areas. Within-area contrasts were largest for NO_2_ and PM_coarse_, while between-area contrasts were the dominant source of variability for the PM_2.5_, PM_2.5_ absorbance, PM_10_, PNC and LDSA [34].

**Table 2:** Distribution of NO_2_, PM_2.5_, PM_2.5_ absorbance, PM_10_, PM_coarse,_ PNC (Particle Number Concentration) and LDSA (Lung Deposited Surface Area) concentrations over the measurement sites

Pollutant	Area(s)	n	Mean	Min	P10	P25	Median	P75	P90	Max
NO_2_ (μg/m^3^)	All: Aarau, Basel, Davos, Geneva, Lugano, Montana, Payerne, Wald	312	21.9	3.7	11.0	13.7	20.5	28.4	35.4	62.9
	Alpine: Davos, Montana	78	18.5	3.7	8.0	11.5	17.0	23.0	30.1	48.7
	Non-alpine: Aarau, Basel, Geneva, Lugano, Payerne, Wald	234	23.0	5.2	11.6	14.1	21.3	29.5	36.2	62.9
	Aarau	40	22.2	11.4	12.9	16.5	20.9	28.9	32.9	35.2
	Basel	40	23.3	11.3	14.3	19.3	22.7	26.9	32.6	39.5
	Davos	38	22.1	7.1	8.0	13.8	20.9	28.6	41.6	48.7
	Geneva	38	29.1	12.0	16.8	21.5	26.0	34.2	47.3	62.9
	Lugano	37	32.5	13.8	20.6	26.0	32.8	36.6	46.6	55.0
	Montana	40	15.0	3.7	7.8	11.1	14.5	18.7	23.1	29.6
	Payerne	40	15.0	8.1	10.1	11.8	13.9	17.0	22.1	34.0
	Wald	39	16.9	5.2	7.0	10.0	13.1	21.9	33.7	48.4
PM_2.5_ (μg/m^3^)	Basel, Geneva, Lugano, Wald	74	14.2	7.8	10.5	12.6	13.5	16.0	17.9	25.1
PM_2.5_ abs (10^−5^ m^−1^)	Basel, Geneva, Lugano, Wald	74	0.94	0.33	0.42	0.66	0.87	1.26	1.49	1.80
PM_10_ (μg/m^3^)	Basel, Geneva, Lugano, Wald	74	20.1	13.0	15.2	17.3	19.3	22.7	26.2	31.9
PM_coarse_ (μg/m^3^)	Basel, Geneva, Lugano, Wald	74	6.1	2.6	3.6	4.5	6.2	7.2	9.0	11.1
PNC (particles/cm^3^)	Basel, Geneva, Lugano, Wald	67	12016	3361	4873	8639	11624	15952	19599	22896
LDSA (μm^2^/cm^3^)	Basel, Geneva, Lugano, Wald	67	32.1	12.2	15.4	24.7	31.4	40.4	46.8	61.3

The area-specific, alpine and non-alpine LUR models and LOOCV validation statistics for NO_2_ are presented in Table 3. Combined-area LUR models for PM_2.5_, PM_2.5_ absorbance, PM_coarse_, PNC and LDSA are shown in Table 4. Pearson correlations between different pollutants are shown in Table 5 for the measured and for the LUR predicted concentrations. Model performance by area and LOAOCV statistics are shown in Additional file 3. Descriptive statistics of the predictor variables used in the models can be found in Additional file 4.

**Table 3:** Alpine, non-alpine and area-specific LUR models for NO_2_

Area(s)	N	Model	Model			Measures of spatial autocorrelation		LOOCV	
			Adj R^2^	R^2^	RMSE	*P*-value of association of residuals with area^a^	Moran’s I (*p*-value)	R^2^	RMSE
Alpine^b^	78	NO_2_ = 7.97 + BUILDINGS_25 * 0.0124 + POP_500 * 0.00658 + TRAFNEAR * 0.000871 + URBGREEN_2000 * -0.00000497	0.50	0.53	6.6	0.1593	0.011 (0.8387)	0.46	7.0
Non-alpine^c^	234	NO_2_ = −0.83 + NO2_2010 * 0.855 + MAJROADLENGTH_25 * 0.201 + HDRES_250 * 0.0000266	0.64	0.65	6.3	**0.0010**	0.0658 (0.2217)	0.63	6.4
Aarau	40	NO_2_ = 2.29 + TRAFLOAD_25 * 0.0000139 + BUILDINGS_75 * 0.0012 + INDUSTRY_5000 * 0.00000332 + MAJROADLENGTH_500 * 0.00179	0.87	0.88	2.7	-	−0.149 (0.1524)	0.84	3.0
Basel	40	NO_2_ = −1.86 + NO2_2010 * 0.738 + HEAVYTRAFLOAD_25 * 0.0019 + HEAVYTRAFLOAD_500 * 0.00000136 + WATER_500 * 0.0000329	0.76	0.78	3.3	-	−0.154 (0.0913)	0.64	4.0
Davos	38	NO_2_ = −6.19 + TRAFLOAD_150 * 0.00000604 + NO2_2010 * 1.63 + ROADLENGTH_50 * 0.0552 + BUILDINGS_25 * 0.0102	0.69	0.73	6.1	-	−0.296 (0.1211)	0.62	6.9
Geneva	38	NO_2_ = 14.2 + POP_2000 * 0.0000987 + MAJROADLENGTH_25 * 0.234 + HDRES_250 * 0.0000619	0.49	0.53	8.3	-	−0.0393 (0.8908)	0.43	8.9
Lugano	37	NO_2_ = 14.1 + TRAFMAJORLOAD_25 * 0.0000293 + TRAFMAJORLOAD_500 * 0.000000331 + WATER_500 * 0.0000436 + INTINVDIST * 0.00357 + INDUSTRY_1000 * 0.0000167	0.64	0.69	5.7	-	−0.0804 (0.4878)	0.57	6.3
Montana	40	NO_2_ = 20.9 + TRAFLOAD_25 * 0.0000183 + LDRES_300 * 0.0000315 + ALT * -0.0143 + BUILDINGS_1000 * 0.000024	0.46	0.52	4.3	-	0.0414 (0.5248)	0.39	4.6
Payerne	40	NO_2_ = 44 + BUILDINGS_50 * 0.00289 + TRAFLOAD_50 * 0.0000126 + ALT * -0.0749	0.61	0.64	3.1	-	0.218 (0.0639)	0.49	3.6
Wald	39	NO_2_ = −10.3 + HEAVYINTINVDIST * 1.35 + NO2_2010 * 1.15 + POP_100 * 0.029	0.89	0.89	3.5	-	−0.00939 (0.8613)	0.86	3.9

^a^Bold = significant association of residuals with study area; ^b^Alpine areas are Davos (*n* = 38) and Montana (*n* = 40); ^c^Non-alpine areas are Aarau (*n* = 40), Basel (*n* = 40), Geneva (*n* = 38), Lugano (*n* = 37), Payerne (*n* = 40) and Wald (*n* = 39)

**Table 4:** Multi-area LUR models for PM_2.5_, PM_2.5_ absorbance, PM_10_, PM_coarse_, PNC and LDSA

Pollutant	N	Model	Model			Measures of spatial autocorrelation		LOOCV	
			Adj R^2^	R^2^	RMSE	*P*-value of association of residuals with area	Moran’s I (*p*-value)	R^2^	RMSE
PM_2.5_ (μg/m^3^)	74	PM_2.5_ = −13.2 + PM25_2010 * 1.81 + MAJROADLENGTH_25 * 0.0478 + URBGREEN_5000 * -0.000000521 + TRAFMAJOR * 0.0000515	0.55	0.57	2.0	0.4530	−0.0558 (0.7222)	0.50	2.2
PM_2.5_ absorbance (10^−5^ m^−1^)	74	PM_2.5_abs = 4.75 + Area_GE * 0.559 + Area_LU * 0.626 + Area_WA * 0.369 + MAJROADLENGTH_25 * 0.00564 + LOG_ALT * -0.715 + HEAVYTRAFLOAD_150 * 0.00000108	0.79	0.81	0.18	1.0000	0.1500 (0.1684)	0.77	0.19
PM_10_ (μg/m^3^)	74	PM_10_ = −19.2 + PM10_2010 * 2.02 + MAJROADLENGTH_25 * 0.0707 + URBGREEN_5000 * -0.00000092	0.62	0.63	2.5	0.1012	0.123 (0.2494)	0.59	2.6
PM_coarse_ (μg/m^3^)	74	PM_coarse_ = −0.69 + PM10_2010 * 0.337 + TRAFMAJORLOAD_75 * 0.000000413 + NATURAL_1000 * -0.00000182 +	0.43	0.45	1.5	0.0551	0.125 (0.242)	0.38	1.6
PNC (particles/cm^3^)	67	PNC = 7805 + Area_GE * 4270 + Area_LU * 5895 + Area_WA * ‘2388 + TRAFLOAD_250 * 0.000110 + ROADLENGTH_100 * 4.26 + MAJROADLENGTH_50 * 19.9 + UGNL_1000 * -0.00273	0.85	0.87	1991	1.0000	−0.0663 (0.7059)	0.82	2255
LDSA (μm^2^/cm^3^)	67	LDSA = 29.9 + Area_GE * 9.17 + Area_LU * 17.3 + Area_WA * 0.502 + MAJROADLENGTH_250 * 0.00317 + ROADLENGTH_100 * 0.0094 + TRAFNEAR * 0.000199 + ALT * -0.0257	0.89	0.91	3.8	1.0000	−0.0434 (0.8349)	0.87	4.2

**Table 5:**
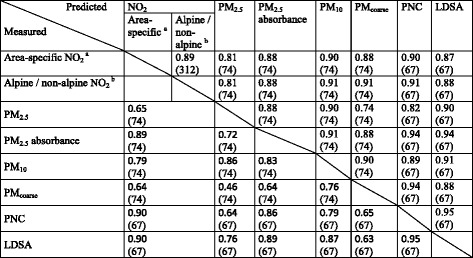
Pearson correlations (n) between different pollutants using measured (lower half) and predicted (upper half) concentrations

^a^Area-specific NO_2_ LUR models were applied to all 312 sites; ^b^Alpine (above 1000 m) and non-alpine (below 1000 m) NO_2_ LUR models were applied to all 312 measurement sites

**Fig. 1:**
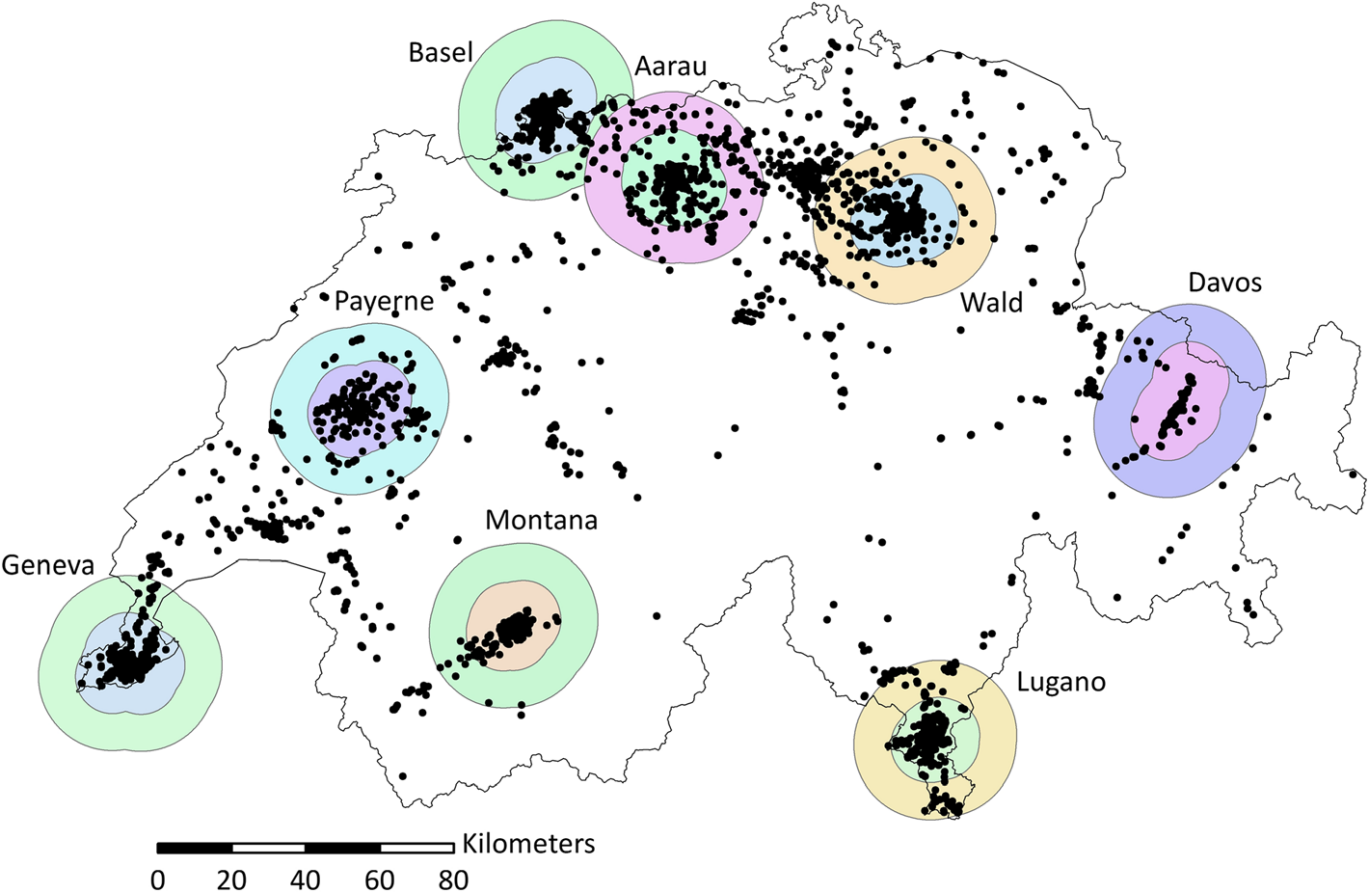
The boundaries of the 10 km and 20 km buffer areas, drawn around the measurement sites which were used to develop the area-specific NO_2_ LUR models. Black dots represent resident locations of SAPALDIA participants

We observed substantial under- and over-prediction by study area, and significant spatial autocorrelation in the residuals for the eight-area NO_2_ model and the four-area PM_2.5_ absorbance, PNC and LDSA models (Additional file 5, Table 2). These models also showed dependence of the model residuals on study area, and substantial over- or under-prediction bias in some of the areas (Additional file 5, Table 3). We therefore ultimately fitted area-specific NO_2_ LUR models which could adequately capture local variability. Additionally, we fitted NO_2_ LUR models for alpine (Davos and Montana) and non-alpine (Aarau, Basel, Geneva, Lugano, Payerne, Wald) areas, which could be applied to predict NO_2_ exposures for addresses outside of the eight SAPALDIA areas, above and below 1000 m, respectively (Fig. 1). For PM_2.5_ absorbance, neither the PM_2.5_ nor the NO_2_ dispersion-model estimates were selected since neither explained the between area variability. For the novel markers of ultrafines (PNC and LDSA), allowing dispersion model estimates for PM_10_ and PM_2.5_ to enter the models resulted in no systematic under- of overprediction by area in the models (Additional file 5, Table 3). However, the inclusion of these dispersion-model estimates did not allow us to capture the spatial variation of ultrafines independently from the mass. To better explain between-area variability in these models, we introduced area-indicators for PM_2.5_ absorbance, PNC and LDSA.

Combined area NO_2_ models for alpine (Davos and Montana) and non-alpine (all others) areas yielded R^2^’s of 0.53 and 0.65, respectively, and performed similarly in cross-validation (LOOCV *R*^*2*^ = 0.46 and 0.63, respectively) (Table 3). Area-specific NO_2_ models yielded an average R^2^ of 0.71 (range 0.52–0.89), which was higher than for the alpine and non-alpine NO_2_ models. LOOCV R^2^ values were on average 10 % (range 3–15 %) lower than model R^2^’s, indicating that models were generally less robust than the multi-area ones. None of the study area specific, nor the alpine or non-alpine NO_2_ models showed significant spatial autocorrelation. While the model residuals remained associated with the study area in the non-alpine model (Table 3), absolute over- and under-prediction for the different areas was small (Additional file 3). The alternative NO_2_ models without regional indicators, with PolluMap NO_2_ estimates, and with area indicators yielded an R^2^’s of 0.52, 0.52 and 0.64, respectively, but could not adequately explain between area variability, or were unable to predict exposures outside of the study areas (Additional file 5).

Multi-area LUR models explained a moderate amount of spatial variance for the different PM mass fractions PM_2.5_ (*R*^*2*^ = 0.57), PM_10_ (*R*^*2*^ = 0.63) and PM_coarse_ (*R*^*2*^ = 0.45). Explained variance was higher for PM_2.5_ absorbance (*R*^*2*^ = 0.81), PNC (*R*^*2*^ = 0.87) and LDSA (*R*^*2*^ = 0.91) (Table 4). For comparison, a model for NO_2_ based on the same four areas yielded a moderate R^2^ of 0.61 in sensitivity analyses (Additional file 5, Table 2). LOOCV R^2^ values were on average 5 % points (range 4–7 %) lower than model R^2^’s for all multi-area models, indicating that models were robust (Table 4). Multi-area models for PM_2.5_, PM_10_ and PM_coarse_ generally performed well in leave-one-area-out cross-validation, predicting similar amounts of within-area spatial variation, whether the area in question was included in the training dataset or not (Additional file 3). However, for all pollutants, the under- or over-prediction of absolute concentrations became more extreme if the area in question was left out from the training dataset (Additional file 3). Alternative models for PNC and LDSA which did not consider and regional indicators were unable to explain between area variability adequately. Alternative models which considered dispersion model predictors yielded high R^2^’s of 0.84 for PNC and 0.89 for LDSA. These models were able to predict between areas, but did not allow us to understand PNC and LDSA variability independently from PM mass. (Additional file 5).

LUR models were successfully applied to available routine monitoring sites and predicted 52 to 83 % of spatial variability for most pollutants (Table 6). Over- and under-prediction was small for NO_2_, PM_2.5_, PM_2.5_ absorbance and PM_10_ compared to the predicted concentration ranges. Over-prediction for PM_coarse_ was larger than for PM_2.5_ and PM_10_, likely because of the increased uncertainty that is introduced when subtracting PM_2.5_ from PM_10_. Sensitivity analysis showed that applying local NO_2_ models within 10 km of the study areas, and alpine and non-alpine models farther outside the study areas, outperformed applying alpine and non-alpine models only, or applying local models within 20 km of the study areas (Additional file 6). While the PNC model performed well in LOOCV, there was no association with the routinely measured averages at fixed monitoring sites. The four available routine monitors generally measured higher numbers of particles than our models predicted, and our PNC model predictions did not capture the spatial contrast measured by the four routine monitors well.

**Table 6:** Results of independent external validation using air pollution data from the routine monitoring sites

Pollutant	*N*	Mean overprediction (standard deviation)	*R* ^*2*^
NO_2_ (μg/m^3^)^a^	102	−2.2 (5.8)	0.75
PM_2.5_ (μg/m^3^)	10	0.090 (1.5)	0.83
PM_2.5_ absorbance (10^−5^ m^−1^)^b,c^	5	−0.13 (0.28)	0.52
PM_10_ (μg/m^3^)	82	0.77 (4.9)	0.71
PM_coarse_ (μg/m^3^)	10	1.2 (1.7)	0.65
PNC (particles/cm^3^)^c^	4	−5058 (17678)	0.00

^a^NO_2_ LUR models were applied to 102 sites in total: the area-specific NO_2_ models were applied to 26 routine monitoring sites within 10 km of the SAPALDIA measurement areas, alpine NO_2_ models were applied to 4 routine monitoring sites outside of SAPALDIA measurement areas, with altitudes above 1000 m, and non-alpine NO_2_ models were applied to 72 routine monitoring sites outside of the SAPALDIA measurement areas, with altitudes below 1000 m (Fig. 1); ^b^The routine monitoring sites measured elemental carbon or soot, but this is known to correlate highly with PM_2.5_ absorbance; ^c^ The PM_2.5_ absorbance and PNC models included area indicators, and were only applied to the sites within 20 km of the SAPALDIA measurement areas

## Discussion

We developed multi-area LUR models for NO_2_, PM_2.5_, PM_2.5_ absorbance, PM_10_, PM_coarse_, PNC and LDSA with moderate to good explained variance across regions of Switzerland. Models for PM_2.5_ absorbance, PNC and LDSA explained the highest amount of spatial variance, and also performed best in LOOCV. Multi-area models for PM_2.5_, PM_10_ and PM_coarse_ adequately captured between-area contrasts, but area-indicators were necessary to adequately capture the between area contrasts for PM_2.5_ absorbance, PNC and LDSA. For NO_2_, study area specific models were preferable to any LUR models which combined areas, since they were able to explain more local spatial variance. However, they were most reliably applied within 10 km of the measurement sites which were used to develop the model. LOAOCV validation for the particulate air pollutant models shows that the multi-area models based on three areas predicted moderate to high amounts of spatial variation in the area which was left out. Validation using independent routine measurement sites shows that the developed models were able to predict moderate to high amounts of spatial variation, except for PNC. For LDSA, we could not verify our LUR model predictions against independent measurements.

LUR models for NO_2_, PM_2.5_, PM_2.5_ absorbance, PM_10_ and PM_coarse_ were previously developed based on measurement campaigns for the SAPALDIA study for the years 1993 and 2003 [10] and for the ESCAPE study for the years 2008 to 2010 [5, 6]. The previous SAPALDIA study also identified that fitting a combined-area model for NO_2_ was challenging because between area variability could not be adequately captured. The models developed in this paper generally performed similarly to previously developed models. Variations in performance may be explained by differences in the site selection procedure, different numbers of sites or the availability of better predictor variables. For a detailed discussion of this comparison and a table summarizing the performance of LUR models from the current and from previous studies in SAPALDIA study areas, we refer to Additional file 7.

There are few LUR models for PNC which can be compared with our model. In Switzerland, only one other LUR model was previously made for PNC [19]. This model was developed for the city of Basel and comprised repeated 20-min measurements at 57–59 measurement sites during three seasons. Median concentrations were log-transformed and a large fraction (50 %) of the (temporal) variability could be explained by the concurrently measured concentration at a nearby urban background site. Subsequently, spatial predictors derived from GIS, and other predictors related to season, meteorology, time and manually observed site characteristics were able to increase the explained variance to 58-68 % (depending on which predictor sets were evaluated) [19]. We cannot directly compare this model for PNC to ours, since it contained temporal as well as spatial terms and used log-transformed median concentrations. In comparison to PNC models from other countries [15–18, 20, 21], which are purely spatial, and used average, not-transformed concentration data, our models performed very well, yielding R^2^ values of 0.84 and 0.89, respectively. The Dutch PNC model for Amsterdam was the only other study based on weeklong observations per site [15], and had a notably higher explained variance (*R*^*2*^ = 0.67, LOOCV *R*^*2*^ = 0.57) than models which were based on shorter-term averages (10–60 min) [16, 18, 19, 21] or mobile monitoring [17, 20].

To understand the spatial predictors of PNC and LDSA independently of particle mass, our main models did not offer PM mass dispersion model estimates. However, in a sensitivity analysis, we allowed the use of dispersion model estimates for PM mass concentrations to enter the models, revealing a strong predictive power of the PM mass dispersion model estimate for the local levels of PNC and LDSA alike (Additional file 5). This may appear counterintuitive, given the rather different small-scale spatial distributions of freshly emitted particles – well indicated by PNC – as compared to the spatially more homogeneous PM_2.5_ and PM_10_. However, we found large spatial variability between study areas for long-term PNC and LDSA measurements [34]. The high predictive ability of dispersion model predictors in PNC and LDSA models and the high correlations in Table 5, show that between-area pollution contrasts are similar for NO_2_, PM mass, PNC and LDSA. The high spatial correlation between NO_2_, PNC and LDSA, which all characterize motor vehicle exhaust in the direct vicinity, was previously discussed in Eeftens et al. [34]. The selection strategy of choosing measurement sites close to residential locations, including side- and back-yards was aimed at capturing the residential variation of PNC and LDSA, rather than the high peaks along busy roads. Therefore, the spatial variability of PNC and LDSA also showed high correlations with other pollutants, such as PM_2.5_ and PM_10_ [34], and it could be predicted by dispersion modelled PM_2.5_ or PM_10_. (Additional file 5).

Variables related to traffic and major roads were selected in nearly all models, mostly in small buffer sizes (25 to 100 m). This has also been observed in earlier studies [5–7], and reflects the major impact of traffic on the adjacent roads on local air quality rather than reflecting the influence of ring roads and motorways. The dispersion model estimates from the Pollumap PM_2.5_ and PM_10_ models are found in the multi-area models for PM_2.5_, PM_10_ and PM_coarse_, and account for a large fraction of the regional and between-area variability (Table 4). These models are too coarse to explain much of the local variability within study areas, and were only included in the area-specific NO_2_ models for Basel, Davos and Wald, but not as the first variable (Table 3). PM_2.5_, PM_10_, PM_coarse_ and PNC models further included large-buffer land-use variables on urban green and natural land, both serving as a sink of pollutants (negative coefficients). These land-use variables do not appear in the multi-area or local NO_2_ models. However, several of the local NO_2_ models include large to medium-size buffers of building density, population density and residential land, representing broader-scale urban activities. In addition, local NO_2_ models include industrial land-use (Aarau and Lugano) indicating local industrial sources. In the Basel and Lugano NO_2_ models, proximity to the Rhine river and Lugano lake was relevant as well. The Rhine above Basel hosts the most important port of Switzerland, and almost all the freight transport ships on the Rhine cross the city. The lake in Lugano is also used by boats, including regular boating services for tourists.

We evaluated model performance in three different ways: 1) using leave-one-out cross validation (LOOCV), which is widely used in previous LUR studies [5–7], 2) leave-one-area-out cross-validation (LOAOCV), as used by a previous study which combined study areas [22] and 3) by independent external validation, where the models were applied to independent sites from a routine measurement network. The second method gives an idea of how well the models predict within-area contrasts in areas which were not used to develop the model. The third method shows how well the models predict independent concentration contrasts at a national level.

Several recent studies pointed out that leave-one-out cross-validation overestimates the true model performance when applied to independent sites, especially if the models were developed based on a limited number of training sites, which increases the risk of over-fitting [39–41]. Our multi-area models are based on a minimum of 67 sites, and our study area specific models on a minimum of 37 sites, increasing the robustness of the models, which is apparent from a small difference between model R^2^ and LOOCV R^2^, as well as good results from LOAOCV and independent external validation.

Independent validation showed that we could predict moderate to large amounts of spatial variability for NO_2_, PM_2.5_, PM_2.5_ absorbance, PM_10_ and PM_coarse_ among routine monitoring sites, which were not used for model development. The comparison of our PNC model with the routine monitoring data must be interpreted with caution. In contrast to all other pollutants, the routine monitoring data for ultrafine particles are derived from different types of monitoring instruments, which measure different particle size ranges, and use optical or electric charge measurement principles. Therefore, the absence of agreement between the LUR model predictions and the routine monitoring for PNC likely highlights the limitation of comparing any metrics of absolute concentrations of ultrafine particles that are derived from multiple different instruments. Hence, this finding should not be interpreted as indicating poor quality of the LUR model.

We minimized the risk of over-fitting by setting a priori criteria for inclusion of variables into our model. These criteria were related to the expected direction of the effect, significance, and distribution of the predictor variables, producing models which were plausible, with coefficients that were robust and not dependent on a small number of sites. Strict selection criteria resulted in the inclusion of 3 to 5 predictors for the local NO_2_, PM_2.5_, PM_10_ and PM_coarse_ models, and 6 to 7 for the PM_2.5_ absorbance, PNC and LDSA models, which included area indicators. Similar screening strategies were used in other studies [43, 44].

While there are no strict rules for a minimum number of sampling sites, for developing LUR models, we observed that the model R^2^ and LOOCV R^2^ were larger for the area-specific NO_2_ models (based on up to 40 sites) and the alpine and non-alpine models than for the model fitted on all eight areas. The risk of over-fitting is larger when using smaller training sets in model building [39–41]. Therefore, we gave preference to the more robust multi-area models for the particulate pollutants, wherever systematic bias in predicting the between-area variability was not an issue. In LOAOCV, we show that the separate alpine and non-alpine NO_2_ models (Additional file 3) are indeed preferable to the NO_2_ model which combined all eight regions (Additional file 5), with lower over- and under-prediction by study area, and better explained variance within each area. In external validation, we further show that the alpine and non-alpine models predict better in the independent external validation (Table 6). The inability to properly model NO_2_ for the Alpine valleys with data from the flatter, much more populated areas of Switzerland and vice versa may be explained by the distinct topographies and meteorological conditions of alpine and non-alpine regions in Switzerland.

While the spatial distributions of different pollutants were already moderately to highly correlated in the SAPALDIA3 measurements, we observed that after applying the LUR models to the measurement sites on which they were fitted, the correlations between pollutants became even higher (Table 5). The LUR models all include different predictors, but these predictors are also correlated. Using these predictors to fit models “smoothes” the random variation in the measurements for all pollutants in a similar way. To not further complicate this challenge, our main models for PNC and LDSA did not allow dispersion modelled PM mass concentrations as predictors. The high correlations between predicted concentrations are a combination of the rather high spatial correlation of these pollutants (lower left part of Table 5) and the artifact of the modelling process. As a consequence, we may expect similarly high correlations when we apply our models to the residential sites of the SAPALDIA cohort participants. It is important to be aware of possible artifacts when using LUR modelled estimates in epidemiological health analyses, especially when fitting 2-pollutant models, for which the collinearity of the estimated exposures will be high. The implication is that epidemiological studies relying on LUR models are limited in disentangling the health effects attributable to the different pollutants, looking at PNC and LDSA independently from PM mass.

Because of the increasing number of LUR models, developed for different study areas, long-term cohort studies such as SAPALDIA have a multitude of exposure estimates available. To avoid multiple testing, we pre-selected models for the health analyses to prevent the use of too many models. Based on our LOAOCV (Additional file 3) and external validation results (Additional file 6), we concluded that long-term exposure to NO_2_ is best estimated by study-area specific models. We will use these models to predict long-term exposure to NO_2_ at the SAPALDIA home and work addresses which fall within 10 km of at least one of the measurement sites used to develop the model for that particular study area. For cohort participants who have moved further beyond the original SAPALDIA study areas, we will estimate the NO_2_ exposure based on the alpine and non-alpine models, respectively, which also performed adequately in external validation. The validity of the models presumably decreases with the distance from the sites used to develop the model. Therefore, an epidemiological sensitivity analysis may address if the effect estimates are affected if those people living further from the measurement sites are excluded. Exposure to PM_2.5_ absorbance, PNC and LDSA model will only be assigned to those coordinates within 20 km from the areas for which they were developed. Exposure to all particulate air pollutants (PM_2.5_, PM_10_ and PM_coarse_) will be assigned to all homes and work addresses by applying the multi-area models developed for these pollutants to all subjects. Since these models are based on measurements made in Basel, Geneva, Lugano and Wald only, it will be necessary to verify in the epidemiological analyses that their application in the remaining four areas, as well as the rest of Switzerland does not affect the relationship with the health outcomes. This might be achieved by performing sensitivity analysis on only those participants who live within 10 km/20 km of the measurement sites which were used to develop the models. Appropriately, the Basel, Geneva, Lugano and Wald study areas are geographically diverse, which suggests the models can be applied in a wide geographical context. Moreover, external validation suggests that throughout Switzerland, the PM_2.5_, PM_10_ and PM_coarse_ models explain moderate to high amounts (*R*^*2*^ = 0.83, 0.71 and 0.65, respectively) of spatial variation.

## Conclusions

We were able to develop a set of LUR models capturing both the between and within-area variability in long-term pollutant concentrations in the study areas of the Swiss SAPALDIA cohort. Multi-area models for PM_2.5_, PM_10_, and PM_coarse_, performed adequately in LOOCV, LOAOCV, and external validation. For PM_2.5_ absorbance, PNC and LDSA, area indicators were needed to capture the between area variance. Model and model performance (evaluated by LOOCV, and for PM_2.5_ absorbance by external validation) was high. For NO_2_, applying study-area specific models was preferable over applying combined-area alpine/non-alpine models. The study-area specific, alpine and non-alpine NO_2_ models, and multi-area models for the particulate air pollutants will be applied to derive exposure of participants of health studies such as the SAPALDIA cohort study. However, we observe high spatial correlations between the estimated pollutants, so the ability to fully disentangle their health effects may remain a challenge.

## Additional files

Additional file 1: Study area descriptions, numbers of sites and measurement periods. (DOCX 17 kb) Additional file 2: Population change in bordering countries of Switzerland over 2000–2011. (DOCX 16 kb) Additional file 3: Explained variance by area and leave-one-area-out cross-validation (LOAOCV) validation. (DOCX 19 kb) Additional file 4: Descriptive statistics of predictor variables used in alpine, non-alpine and area-specific NO_2_ LUR models, and (continued) in multi-area PM_2.5_, PM_2.5_ absorbance, PM_10_, PM_coarse_, PNC and LDSA LUR models. (DOCX 25 kb) Additional file 5: Process of model selection and description and evaluation of alternative LUR models, which were not eventually selected. (DOCX 34 kb) Additional file 6: Results of independent external validation for NO_2_, using different cut-off distances for the application of local versus alpine/non-alpine LUR models (*n* = 102). (DOCX 14 kb) Additional file 7: Overview of model performance of LUR models built previously for Swiss study areas. (DOCX 60 kb)

## Abbreviations

CORINE: COoRdination of INformation on the Environment
GIS: Geographic information systems
LDSA: lung deposited surface area
LOAOCV: Leave-one-area-out cross-validation
LOOCV: leave-one-out cross-validation
LUR: land use regression
NO_2_: nitrogen dioxide
NO_X_: nitrogen oxides
PM_2.5_: mass concentration of particles less than 2.5 μm in size
PM_10_: mass concentration of particles less than 10 μm in size
PM_2.5_ absorbance: measurement of the blackness of PM_2.5_ filters; a proxy for elemental carbon, which is the dominant light absorbing substance
PM_coarse_: mass concentration of particles between 2.5 μm and 10 μm in size
PNC: particle number concentration
