# Supplementary material for: Development of land use regression models for nitrogen dioxide, ultrafine particles, lung deposited surface area, and four other markers of particulate matter pollution in the Swiss SAPALDIA regions
Source: Environ Health. 2016 Apr 18;15:53. doi: 10.1186/s12940-016-0137-9 (PMC4835865; doi:10.1186/s12940-016-0137-9)
Supplement: Additional file 1: — Study area descriptions, numbers of sites and measurement periods. (DOCX 17 kb) [file 12940_2016_137_MOESM1_ESM.docx]

Additional file 1: Study area descriptions, numbers of sites and measurement periods.

| Area(s) | Description | Number of sites by pollutant | | | | | | | Periods in which original measurements took place ^a^ | | |
| --- | --- | --- | --- | --- | --- | --- | --- | --- | --- | --- | --- |
|  |  | NO_2_ | PM_2.5_ | PM_2.5_abs | PM_10_ | PM_coarse_ | PNC | LDSA | Winter | Intermediate | Summer |
| Aarau | Medium city | 40 |  |  |  |  |  |  | Jan-Feb 2012 | Mar-Apr 2012 | Jun 2012 |
| Basel | Major city | 40 | 20 | 20 | 20 | 20 | 17 | 17 | Jan-Feb 2011 | Mar-Apr 2011 | May-Jun  2011 |
| Davos | Alpine area | 38 |  |  |  |  |  |  | Nov-Dec 2011 | May 2011 | Aug-Sep  2011 |
| Geneva | Major city | 38 | 18 | 18 | 18 | 18 | 16 | 16 | Nov-Dec 2011 | Apr-May 2011 | Aug-Sep  2011 |
| Lugano | Medium city | 37 | 17 | 17 | 17 | 17 | 16 | 16 | Jan-Feb 2012 | Mar-Apr 2012 | May-Jun 2012 |
| Montana | Alpine area | 40 |  |  |  |  |  |  | Nov-Dec 2012 | May 2012 | Aug-Sep 2012 |
| Payerne | Rural area | 40 |  |  |  |  |  |  | Jan-Feb 2011 | Mar-Apr 2011 | Jun 2011 |
| Wald | Rural area | 39 | 19 | 19 | 19 | 19 | 18 | 18 | Nov-Dec 2012 | Apr-May 2012 | Aug-Sep 2012 |
| All areas (8) | Aarau, Basel, Davos, Geneva, Lugano, Montana, Payerne, Wald | 312 | 74 | 74 | 74 | 74 | 67 | 67 |  |  |  |
| Alpine areas (2) | Davos, Montana | 78 |  |  |  |  |  |  |  |  |  |
| Non-alpine areas (6) | Aarau, Basel, Geneva, Lugano, Payerne, Wald | 234 | 74 | 74 | 74 | 74 | 67 | 67 |  |  |  |
| Pooled NO_2_+PM+UFP areas (4) ^b^ | Basel, Geneva, Lugano, Wald | 154 | 74 | 74 | 74 | 74 | 67 | 67 |  |  |  |

^a^ Original measurement periods are indicative. Due to limited availability of equipment, only a limited number of sites could be measured simultaneously. Any bias introduced by temporal variability between measurement periods was corrected by a correction factor derived from continuous measurements at an area-specific reference site which sampled during all measurement periods. For the modelling, we used the long-term corrected bi-annual mean concentrations, which are more comparable between areas. The details of how these were derived were published previously by Eeftens et al (2015).

^b^ Here we mean all areas where NO_2_, PM and UFP were measured; some areas had only NO_2_.

Eeftens M, Phuleria HC, Meier R, Aguilera I, Corradi E, Davey M, Ducret-Stich R, Fierz M, Gehrig R, Ineichen A: **Spatial and temporal variability of ultrafine particles, NO 2, PM 2.5, PM 2.5 absorbance, PM 10 and PM coarse in Swiss study areas**. *Atmospheric Environment* 2015, **111**:60-70.
