# Supplementary material for: Development of land use regression models for nitrogen dioxide, ultrafine particles, lung deposited surface area, and four other markers of particulate matter pollution in the Swiss SAPALDIA regions
Source: Environ Health. 2016 Apr 18;15:53. doi: 10.1186/s12940-016-0137-9 (PMC4835865; doi:10.1186/s12940-016-0137-9)
Supplement: Additional file 2: — Population change in bordering countries of Switzerland over 2000–2011. (DOCX 16 kb) [file 12940_2016_137_MOESM2_ESM.docx]

Additional file 2: Population change in bordering countries of Switzerland over 2000-2011

| **Country** | **Population 2000** | **Population 2011** | **Growth** |
| --- | --- | --- | --- |
| Austria | 8011566 | 8423635 | 1.051 |
| France | 60911057 | 65371613 | 1.073 |
| Germany | 82211508 | 81797673 | 0.995 |
| Italy | 56942108 | 60723569 | 1.066 |
| **Average** |  |  | **1.047** |

Data source: http://data.worldbank.org/indicator/SP.POP.TOTL.
