# Supplementary material for: Development of land use regression models for nitrogen dioxide, ultrafine particles, lung deposited surface area, and four other markers of particulate matter pollution in the Swiss SAPALDIA regions
Source: Environ Health. 2016 Apr 18;15:53. doi: 10.1186/s12940-016-0137-9 (PMC4835865; doi:10.1186/s12940-016-0137-9)
Supplement: Additional file 3: — Explained variance by area and leave-one-area-out cross-validation (LOAOCV) validation. (DOCX 19 kb) [file 12940_2016_137_MOESM3_ESM.docx]

Additional file 3: Explained variance by area and leave-one-area-out cross-validation (LOAOCV) validation

| Model | Area | N | Explained variance by area, based on full model ^a^ | | | Explained variance by area, based on leave-one-area-out cross validation ^b^ | | |
| --- | --- | --- | --- | --- | --- | --- | --- | --- |
|  |  |  | Over-prediction  (p-value) | R² | RMSE | Over-prediction (p-value) | R² | RMSE |
| Alpine NO_2_ (µg/m³) (n=78) | DA | 38 | -1.4 (0.1688) | 0.61 | 7.0 | **-6.1 (0.0001)** | 0.54 | 7.6 |
|  | MO | 40 | 1.4 (0.1797) | 0.32 | 4.8 | 2.5 (0.0836) | 0.33 | 4.8 |
| Non-alpine NO_2_ (µg/m³) (n=234) | AR | 40 | **2.8 (0.0031)** | 0.74 | 3.8 | **4.1 (<0.0001)** | 0.73 | 3.9 |
|  | BS | 40 | **2.3 (0.0144)** | 0.50 | 4.8 | **2.9 (0.0037)** | 0.49 | 4.8 |
|  | GE | 38 | -1.5 (0.1355) | 0.45 | 8.7 | -1.8 (0.0696) | 0.45 | 8.8 |
|  | LU | 37 | -1.6 (0.1164) | 0.40 | 7.5 | **-2 (0.0471)** | 0.37 | 7.7 |
|  | PA | 40 | -0.7 (0.4798) | 0.40 | 4.0 | -1.2 (0.2277) | 0.39 | 4.0 |
|  | WA | 39 | -1.7 (0.0738) | 0.81 | 4.5 | **-2.4 (0.0165)** | 0.81 | 4.5 |
| Multi-area PM_2.5_ (µg/m³) (n=74) | BS | 20 | 0.5 (0.2925) | 0.29 | 1.8 | 0.7 (0.1284) | 0.18 | 1.9 |
|  | GE | 18 | 0.2 (0.6515) | 0.33 | 1.3 | **1.7 (0.0012)** | 0.34 | 1.3 |
|  | LU | 17 | -0.5 (0.3292) | 0.25 | 2.4 | **-1.8 (0.0009)** | 0.24 | 2.4 |
|  | WA | 19 | -0.3 (0.5503) | 0.10 | 2.4 | **-1.2 (0.0132)** | 0.1 | 2.4 |
| Multi-area PM_2.5_ absorbance with area indicators (10^-5^ m^-1^) (n=74) | BS | 20 | 0.0 (1.000) | 0.57 | 0.095 |  |  |  |
|  | GE | 18 | 0.0 (1.000) | 0.50 | 0.21 |  |  |  |
|  | LU | 17 | 0.0 (1.000) | 0.36 | 0.22 |  |  |  |
|  | WA | 19 | 0.0 (1.000) | 0.29 | 0.18 |  |  |  |
| Multi-area PM_10_ (µg/m³) (n=74) | BS | 20 | **1.1 (0.0389)** | 0.06 | 1.6 | **1.6 (0.0073)** | 0.05 | 1.6 |
|  | GE | 18 | -0.1 (0.8265) | 0.36 | 2.3 | 0.4 (0.4734) | 0.36 | 2.3 |
|  | LU | 17 | -1.1 (0.063) | 0.26 | 2.9 | **-3 (<0.0001)** | 0.29 | 2.8 |
|  | WA | 19 | -0.1 (0.8741) | 0.21 | 2.6 | -0.4 (0.4588) | 0.21 | 2.6 |
| Multi-area PM_coarse_ (µg/m³) (n=74) | BS | 20 | **0.8 (0.0195)** | 0.02 | 1.4 | **1.2 (0.0007)** | 0.04 | 1.4 |
|  | GE | 18 | -0.3 (0.3842) | 0.17 | 1.6 | -0.4 (0.2846) | 0.18 | 1.6 |
|  | LU | 17 | -0.4 (0.2915) | 0.34 | 1.6 | **-1.1 (0.0022)** | 0.24 | 1.7 |
|  | WA | 19 | -0.2 (0.5537) | 0.35 | 1.1 | **-1 (0.0046)** | 0.35 | 1.1 |
| Multi-area PNC with area indicators (particles/cm³) (n=67) | BS | 17 | 0.0 (1.000) | 0.24 | 913 |  |  |  |
|  | GE | 16 | 0.0 (1.000) | 0.66 | 2451 |  |  |  |
|  | LU | 16 | 0.0 (1.000) | 0.66 | 1949 |  |  |  |
|  | WA | 18 | 0.0 (1.000) | 0.47 | 1827 |  |  |  |
| Multi-area LDSA with area indicators (µm²/cm³) (n=67) | BS | 17 | 0.0 (1.000) | 0.63 | 1.5 |  |  |  |
|  | GE | 16 | 0.0 (1.000) | 0.62 | 3.8 |  |  |  |
|  | LU | 16 | 0.0 (1.000) | 0.48 | 4.9 |  |  |  |
|  | WA | 18 | 0.0 (1.000) | 0.47 | 4.0 |  |  |  |

^a^ Predictions were made using the full model based on all available sites and applied to each area individually; ^b^ Predictions were made using the full model minus one area, while keeping the same predictors in the model, and letting the coefficients vary. This model was then applied to the left-out area. The models for PM_2.5_ absorbance, PNC and LDSA, which included area indicators, could not be applied to the other areas.
