# Supplementary material for: Development of land use regression models for nitrogen dioxide, ultrafine particles, lung deposited surface area, and four other markers of particulate matter pollution in the Swiss SAPALDIA regions
Source: Environ Health. 2016 Apr 18;15:53. doi: 10.1186/s12940-016-0137-9 (PMC4835865; doi:10.1186/s12940-016-0137-9)
Supplement: Additional file 4: — Descriptive statistics of predictor variables used in alpine, non-alpine and area-specific NO2 LUR models, and (continued) in multi-area PM2.5, PM2.5 absorbance, PM10, PMcoarse, PNC and LDSA LUR models. (DOCX 25 kb) [file 12940_2016_137_MOESM4_ESM.docx]

Additional file 4: Descriptive statistics of predictor variables used in alpine, non-alpine and area-specific NO_2_ LUR models, and (continued) in multi-area PM_2.5_, PM_2.5_ absorbance, PM_10_, PM_coarse_, PNC and LDSA LUR models

| Area | N |  | Mean | Minimum | P10 | P25 | Median | P75 | P90 | Maximum |
| --- | --- | --- | --- | --- | --- | --- | --- | --- | --- | --- |
| Alpine ^a^ | 78 | BUILDINGS_25 (m²) | 288 | 0 | 0 | 76.9 | 240 | 420 | 689 | 913 |
|  |  | POP_500 (number) | 1061 | 46.9 | 185 | 571 | 928 | 1609 | 2025 | 2502 |
|  |  | TRAFNEAR (mvh / 24h) | 2016 | 70 | 140 | 170 | 925 | 2456 | 6092 | 8910 |
|  |  | URBGREEN_2000 (m²) | 363546 | 0 | 0 | 52534 | 370007 | 534038 | 860124 | 860124 |
| Non-  alpine ^b^ | 234 | NO2_2010 (µg/m³) | 24.4 | 10.7 | 15.9 | 18.3 | 25.7 | 29.8 | 32.2 | 38.7 |
|  |  | MAJROADLENGTH_25 (m) | 11.3 | 0 | 0 | 0 | 0 | 19.4 | 47.4 | 50.6 |
|  |  | HDRES_250 (m²) | 26441 | 0 | 0 | 0 | 0 | 3911 | 130031 | 196349 |
| AR | 40 | TRAFLOAD_25 (mvh/24h*m) | 323051 | 0 | 3309 | 23712 | 147776 | 574857 | 882876 | 1043111 |
|  |  | BUILDINGS_75 (m²) | 3724 | 0 | 1855 | 2910 | 3681 | 4681 | 5472 | 7075 |
|  |  | INDUSTRY_5000 (m²) | 2576379 | 1592444 | 1905296 | 2470178 | 2596364 | 2776687 | 2981011 | 3566745 |
|  |  | MAJROADLENGTH_500 (m) | 1327 | 0 | 0 | 701 | 1058 | 1852 | 3141 | 4059 |
| BS | 40 | NO2_2010 (µg/m³) | 27.6 | 19.5 | 21.8 | 26.1 | 28.5 | 29.8 | 31.3 | 33.8 |
|  |  | HEAVYTRAFLOAD_25 (mvh/24h*m) | 1466 | 0 | 0 | 81.1 | 858 | 2269 | 4127 | 7550 |
|  |  | HEAVYTRAFLOAD_500 (mvh/24h*m) | 1143366 | 35549 | 110685 | 204267 | 616039 | 1226274 | 3439149 | 5434635 |
|  |  | WATER_500 (m²) | 15810 | 0 | 0 | 0 | 0 | 0 | 70213 | 196011 |
| DA | 38 | TRAFLOAD_150 (mvh/24h*m) | 783777 | 9486 | 41912 | 201043 | 563325 | 1367862 | 1780339 | 2881598 |
|  |  | NO2_2010 (µg/m³) | 8.09 | 3.8 | 5 | 6.7 | 8.7 | 9.5 | 10 | 10.6 |
|  |  | ROADLENGTH_50 (m) | 126 | 0 | 91.5 | 99.3 | 118 | 163 | 175 | 252 |
|  |  | BUILDINGS_25 (m²) | 337 | 0 | 0 | 93.6 | 300 | 534 | 794 | 913 |
| GE | 38 | POP_2000 (number) | 95664 | 12160 | 48817 | 71266 | 98010 | 124159 | 152907 | 166244 |
|  |  | MAJROADLENGTH_25 (m) | 17.4 | 0 | 0 | 0 | 0 | 45.2 | 48.3 | 49.9 |
|  |  | HDRES_250 (m²) | 22624 | 0 | 0 | 0 | 0 | 0 | 84866 | 180988 |
| LU | 37 | TRAFMAJORLOAD_25 (mvh/24h*m) | 104018 | 0 | 0 | 0 | 0 | 226782 | 379031 | 468472 |
|  |  | TRAFMAJORLOAD_500 (mvh/24h*m) | 35868328 | 1879722 | 15490527 | 27063309 | 32449538 | 45422352 | 59526049 | 76098539 |
|  |  | WATER_500 (m²) | 22107 | 0 | 0 | 0 | 0 | 0 | 136396 | 204134 |
|  |  | INTINVDIST (mvh/24h*m^-1^) | 489 | 1.66 | 9.72 | 64.4 | 177 | 567 | 1325 | 3484 |
|  |  | INDUSTRY_1000 (m²) | 48962 | 0 | 0 | 0 | 0 | 0 | 178071 | 480129 |
| MO | 40 | TRAFLOAD_25 (mvh/24h*m) | 136062 | 1524 | 6911 | 16502 | 94955 | 177464 | 424590 | 474993 |
|  |  | LDRES_300 (m²) | 205877 | 17242 | 126175 | 168051 | 216247 | 252322 | 273565 | 282742 |
|  |  | ALT (m) | 1335 | 906 | 1107 | 1191 | 1408 | 1467 | 1505 | 1661 |
|  |  | BUILDINGS_1000 (m²) | 173948 | 59288 | 74081 | 101882 | 160770 | 249525 | 281589 | 294677 |
| PA | 40 | BUILDINGS_50 (m²) | 1164 | 0 | 162 | 582 | 939 | 1560 | 2256 | 4403 |
|  |  | TRAFLOAD_50 (mvh/24h*m) | 168959 | 0 | 9008 | 20947 | 102715 | 316507 | 396844 | 573983 |
|  |  | ALT (m) | 460 | 447 | 449 | 449 | 451 | 468 | 478 | 511 |
| WA | 39 | HEAVYINTINVDIST (mvh/24h*m^-1^) | 3.59 | 0.0374 | 0.18 | 0.297 | 1.07 | 5.47 | 13.2 | 18.3 |
|  |  | NO2_2010 (µg/m³) | 17.3 | 10.7 | 12.9 | 15 | 17.7 | 19.6 | 21.6 | 22.7 |
|  |  | POP_100 (number) | 86.1 | 0.0247 | 13.6 | 32.4 | 85.3 | 142 | 161 | 223 |

^a^ alpine areas are Davos and Montana; ^b^ non-alpine areas are Aarau, Basel, Geneva, Lugano, Payerne and Wald

Additional file 4(continued): Descriptive statistics of predictor variables used in alpine, non-alpine and area-specific NO_2_ LUR models, and (continued) in multi-area PM_2.5_, PM_2.5_ absorbance, PM_10_, PM_coarse_, PNC and LDSA LUR models

| Pollutant | N | Variable | Mean | Minimum | P10 | P25 | Median | P75 | P90 | Maximum |
| --- | --- | --- | --- | --- | --- | --- | --- | --- | --- | --- |
| PM_2.5_ | 74 | PM25_2010 (µg/m³) | 15.2 | 12.3 | 13.4 | 14 | 15.2 | 15.9 | 17.4 | 18.3 |
|  |  | MAJROADLENGTH_25 (m) | 6.65 | 0 | 0 | 0 | 0 | 0 | 32.7 | 49.4 |
|  |  | URBGREEN_5000 (m²) | 1971904 | 0 | 0 | 0 | 2010248 | 2650733 | 3988893 | 5087843 |
|  |  | TRAFMAJOR (mvh/24h) | 13059 | 5007 | 5350 | 7960 | 10530 | 16089 | 24948 | 40495 |
| PM_2.5_ abs | 74 | MAJROADLENGTH_25 (m) | 6.65 | 0 | 0 | 0 | 0 | 0 | 32.7 | 49.4 |
|  |  | LOG_ALT (m) | 5.96 | 5.52 | 5.58 | 5.64 | 5.93 | 6.23 | 6.48 | 6.73 |
|  |  | HEAVYTRAFLOAD_150 (mvh/24h*m) | 45739 | 0 | 2684 | 10259 | 23384 | 48268 | 120651 | 291701 |
| PM_10_ | 74 | PM10_2010 (µg/m³) | 20.1 | 15.8 | 17.5 | 18.6 | 20.2 | 21.8 | 22.4 | 23.9 |
|  |  | MAJROADLENGTH_25 (m) | 6.65 | 0 | 0 | 0 | 0 | 0 | 32.7 | 49.4 |
|  |  | URBGREEN_5000 (m²) | 1971904 | 0 | 0 | 0 | 2010248 | 2650733 | 3988893 | 5087843 |
| PM_coarse_ | 74 | PM10_2010 (µg/m³) | 20.1 | 15.8 | 17.5 | 18.6 | 20.2 | 21.8 | 22.4 | 23.9 |
|  |  | TRAFMAJORLOAD_75 (mvh/24h*m) | 665338 | 0 | 0 | 0 | 0 | 1018514 | 2225218 | 6005253 |
|  |  | NATURAL_1000 (m²) | 163559 | 0 | 0 | 0 | 0 | 264750 | 581360 | 956116 |
| PNC | 67 | TRAFLOAD_250 (mvh/24h*m) | 8655104 | 140390 | 948200 | 2188485 | 6541464 | 11878725 | 24039139 | 49085068 |
|  |  | ROADLENGTH_100 (m) | 399 | 0 | 135 | 268 | 384 | 539 | 637 | 880 |
|  |  | MAJROADLENGTH_50 (m) | 22.4 | 0 | 0 | 0 | 0 | 0 | 95.6 | 163 |
|  |  | UGNL_1000 (m²) | 247557 | 0 | 0 | 0 | 149198 | 444269 | 664392 | 956116 |
| LDSA | 67 | MAJROADLENGTH_250 (m) | 555 | 0 | 0 | 0 | 483 | 825 | 1464 | 2106 |
|  |  | ROADLENGTH_100 (m) | 399 | 0 | 135 | 268 | 384 | 539 | 637 | 880 |
|  |  | TRAFNEAR (mvh/24h) | 3875 | 8 | 90 | 170 | 720 | 3170 | 11920 | 40495 |
|  |  | ALT (m) | 417 | 250 | 274 | 283 | 375 | 511 | 670 | 834 |
