# Supplementary material for: Development of land use regression models for nitrogen dioxide, ultrafine particles, lung deposited surface area, and four other markers of particulate matter pollution in the Swiss SAPALDIA regions
Source: Environ Health. 2016 Apr 18;15:53. doi: 10.1186/s12940-016-0137-9 (PMC4835865; doi:10.1186/s12940-016-0137-9)
Supplement: Additional file 5: — Process of model selection and description and evaluation of alternative LUR models, which were not eventually selected. (DOCX 34 kb) [file 12940_2016_137_MOESM5_ESM.docx]

Additional file 5: Process of model selection and description and evaluation of alternative LUR models, which were not eventually selected

Table 1: Steps followed in the selection of the models for the different pollutants

| Pollutant | Model 1 | Model 2 | Model 3 | Model 4 |
| --- | --- | --- | --- | --- |
| NO_2_ | N=312, 8 areas  Considering NO2 PolluMap (NO2_2010 not selected)  Not considering area indicators  Issue: Residual spatial autocorrelation | N=312, 8 areas  Forcing in NO_2_ Pollumap (NO2_2010 forced in as first variable)  Not considering area indicators  Issue: Residual spatial autocorrelation, variable NO2_2010 insignificant | N=312, 8 areas  Not considering NO_2_ Pollumap  Considering area indicators  Issue: not possible to apply outside of study areas | Single area models (N=≤40 each)  Alpine model (N=78)  Non-alpine model (N=234)  Considering NO_2_ Pollumap  Not considering area indicators |
| Extra 4 Area NO_2_ model (developed only for comparison purposes) | N=154, 4 areas  Considering NO2 PolluMap (NO2_2010 selected)  Not considering area indicators |  |  |  |
| PM_2.5_ | N=74, 4 areas  Considering PM_2.5_ Pollumap (PM25_2010 selected)  Not considering area indicators |  |  |  |
| PM_2.5_ absorbance | N=74, 4 areas  Considering PM_2.5_ Pollumap (PM25_2010 selected)  Not considering area indicators  Issue: Residual spatial autocorrelation | N=74, 4 areas  Considering NO_2_ Pollumap (NO2_2010 selected)  Not considering area indicators  Issue: Residual spatial autocorrelation | N=74, 4 areas  Not considering NO_2_ Pollumap  Considering area indicators |  |
| PM10 | N=74, 4 areas  Considering PM_10_ Pollumap (PM10_2010 selected)  Not considering area indicators |  |  |  |
| PMcoarse | N=74, 4 areas  Considering PM_10_ Pollumap (PM10_2010 selected)  Not considering area indicators |  |  |  |
| PNC | N=67, 4 areas Not considering PolluMap  Not considering area indicators Issue: Residual spatial autocorrelation | N=67, 4 areas Considering PolluMap PM_2.5_ and PM_10_ (PM10_2010 selected)  Not considering area indicators Issue: Models did not allow understanding LDSA separately from PM mass | N=67, 4 areas  Not considering NO_2_ Pollumap  Considering area indicators |  |
| LDSA | N=67, 4 areas Not considering PolluMap  Not considering area indicators Issue: Residual spatial autocorrelation | N=67, 4 areas Considering PolluMap PM_2.5_ and PM_10_ (PM25_2010 selected)  Not considering area indicators Issue: Models did not allow understanding LDSA separately from PM mass | N=67, 4 areas  Not considering NO_2_ Pollumap  Considering area indicators |  |

Green=this is the model that was ultimately selected, Yellow=this was the main reason not to choose this model.

Table 2: Descriptions of alternative LUR models, which were not eventually selected

| Pollutant (unit, N) | Model | Model | | | Measures of spatial  autocorrelation | | | LOOCV | |
| --- | --- | --- | --- | --- | --- | --- | --- | --- | --- |
|  |  | Adj R² | R² | RMSE | Association  of residuals  with area | Moran’s I  (p-value) | Geary’s C  (p-value) | R² | RMSE |
| NO_2_ model 1  (µg/m³, n=312) | NO_2_ = 9.60 +  MAJROADLENGTH_500 * 0.000873 +  TRAFLOAD_25 * 0.0000163 +  BUILDINGS_250 * 0.0000976 +  HDRES_1000 * 0.00000631 +  LDRES_500 * 0.00000696 | 0.51 | 0.52 | 7.29 | **<0.0001** | **0.401 (<0.0001)** | 0.934  (0.532) | 0.50 | 7.4 |
| NO_2_ model 2  (µg/m³, n=312) | NO_2_ = 7.79 +  NO2_2010 * 0.146 +  TRAFLOAD_25 * 0.0000168 +  BUILDINGS_200 * 0.000152 +  HDRES_1000 * 0.00000679 +  LDRES_500 * 0.00000651 | 0.51 | 0.52 | 7.30 | **<0.0001** | **0.436 (<0.0001)** | 0.907 (0.3775) | 0.49 | 7.4 |
| NO_2_ model 3  (µg/m³, n=312) | NO_2_ =  Area_AR * 121 + Area_BS * 115 +  Area_DA * 151 + Area_GE * 127 +  Area_LU * 129 + Area_MO * 141 +  Area_PA * 122 + Area_WA * 129 +  TRAFLOAD_25 * 0.0000182 +  BUILDINGS_250 * 0.000159 +  LOG_ALT * -18.4 | 0.62 | 0.64 | 6.39 | 1.0000 | 0.052  (0.3236) | 1.168  (0.1088) | 0.61 | 6.6 |
| Extra NO_2_ model 1 for 4 areas  (µg/m³, n=154) | NO_2_ = -3.45 +  NO2_2010 * 0.999 +  MAJORROADLENGTH_25 * 0.233 | 0.61 | 0.61 | 7.02 | **0.0162** | 0.053  (0.2750) | 0.958  (0.7388) | 0.60 | 7.2 |
| PM_2.5_ absorbance model 1  (10^-5^ m^-1^, n=74) | PM_2.5_ abs = -2.11 +  PM25_2010 * 0.195 +  MAJROADLENGTH_25 * 0.00669 +  HEAVYTRAFLOAD_150 * 0.00000118 | 0.77 | 0.78 | 0.192 | 0.0931 | **0.279**  **(0.0134)** | 0.881  (0.5798) | 0.74 | 0.203 |
| PM_2.5_ absorbance model 2  (10^-5^ m^-1^, n=74) | PM_2.5_ abs = -0.0698 +  NO2_2010 * 0.0379 +  MAJROADLENGTH_25 * 0.00750 | 0.65 | 0.66 | 0.235 | **<0.0001** | **0.486**  **(<0.0001)** | **0.529**  **(0.0279)** | 0.63 | 0.244 |
| PNC model 1  (particles/cm³, n=67) | PNC = 7081 +  MAJROADLENGTH_1000 * 0.196 +  NATURAL_1000 * -0.0047 +  TRAFLOAD_75 * 0.00136 +  HDRES_250 * 0.0192 +  LDRES_2000 * 0.000465 | 0.74 | 0.76 | 2647 | **<0.0001** | **0.315**  **(0.015)** | 0.793  (0.3896) | 0.72 | 2755 |
| PNC model 2  (particles/cm³, n=67) | PNC = -22499 +  PM10_2010 * 1711 +  MAJROADLENGTH_75 * 16.1 +  NATURAL_1000 * -0.00333 | 0.83 | 0.84 | 2140 | 0.1050 | -0.175  (0.2386) | 1.135 (0.5767) | 0.82 | 2254 |
| LDSA model 1  (µm²/cm³, n=67) ^d^ | LDSA = 39.1 +  NATURAL_1000 * -0.0000121 +  HDRES_300 * 0.0000372 +  MAJROADLENGTH_50 * 0.0378 +  ALT * -0.0217 +  TRAFMAJORLOAD_200 * 0.000000397 | 0.70 | 0.72 | 6.4 | **<0.0001** | **0.491**  **(0.0002)** | 0.612  (0.1074) | 0.66 | 6.8 |
| LDSA model 2  (µm²/cm³, n=67) | LDSA = -60.8 +  PM25_2010 * 6.09 +  TRAFLOAD_75 * 0.00000202 +  NATURAL_1000 * -0.00000629 | 0.88 | 0.89 | 4.0 | 0.7875 | -0.0817  (0.6236) | 1.3382 (0.1606) | 0.87 | 4.2 |

Table 3: Explained variance by area and leave-one-area-out cross-validation (LOAOCV) for alternative LUR models which were not eventually selected

| Model | Area | N | Explained variance by area,  based on full model ^a^ | | | Explained variance by area, based on leave-one-area-out cross validation ^b^ | | |
| --- | --- | --- | --- | --- | --- | --- | --- | --- |
|  |  |  | Over-prediction  (p-value) | R² | RMSE | Over-prediction (p-value) | R² | RMSE |
| NO_2_ model 1  (µg/m³, n=312) | AR | 40 | 1.7 (0.1019) | 0.76 | 3.6 | **2.5 (0.0239)** | 0.74 | 3.8 |
|  | BS | 40 | **3.4 (0.0013)** | 0.32 | 5.5 | **5.3 (<0.0001)** | 0.29 | 5.7 |
|  | DA | 38 | **-5.2 (<0.0001)** | 0.51 | 7.8 | **-6.9 (<0.0001)** | 0.51 | 7.8 |
|  | GE | 38 | -1.5 (0.1642) | 0.39 | 9.2 | -1.1 (0.3184) | 0.38 | 9.3 |
|  | LU | 37 | **-4.2 (0.0002)** | 0.31 | 8.1 | **-8.7 (<0.0001)** | 0.38 | 7.6 |
|  | MO | 40 | **2.7 (0.0097)** | 0.33 | 4.8 | **3.4 (0.0023)** | 0.33 | 4.8 |
|  | PA | 40 | **2.4 (0.0223)** | 0.42 | 3.9 | **3.0 (0.0058)** | 0.41 | 3.9 |
|  | WA | 39 | 0.0 (0.9969) | 0.75 | 5.2 | 0.2 (0.8666) | 0.75 | 5.2 |
| NO_2_ model 2  (µg/m³, n=312) | AR |  | **2.5 (0.0177)** | 0.74 | 3.8 | **3.6 (0.0008)** | 0.74 | 3.8 |
|  | BS |  | **3.6 (0.0006)** | 0.29 | 5.7 | **5.2 (<0.0001)** | 0.27 | 5.8 |
|  | DA |  | **-5.9 (<0.0001)** | 0.52 | 7.7 | **-10.9 (<0.0001)** | 0.57 | 7.3 |
|  | GE |  | -2.0 (0.0623) | 0.42 | 9.0 | **-2.5 (0.0215)** | 0.42 | 8.9 |
|  | LU |  | **-4.1 (0.0002)** | 0.33 | 7.9 | **-8.8 (<0.0001)** | 0.38 | 7.6 |
|  | MO |  | 1.9 (0.0669) | 0.38 | 4.6 | **3.0 (0.0057)** | 0.37 | 4.7 |
|  | PA |  | **2.9 (0.0006)** | 0.46 | 3.8 | **3.4 (0.0014)** | 0.45 | 3.8 |
|  | WA |  | 0.4 (0.6806) | 0.75 | 5.2 | 0.74 (0.4934) | 0.76 | 5.1 |
| NO_2_ model 3  (µg/m³, n=312) | AR | 40 | 0.0 (1.000) | 0.79 | 3.4 |  |  |  |
|  | BS | 40 | 0.0 (1.000) | 0.35 | 5.4 |  |  |  |
|  | DA | 38 | 0.0 (1.000) | 0.48 | 8.0 |  |  |  |
|  | GE | 38 | 0.0 (1.000) | 0.39 | 9.2 |  |  |  |
|  | LU | 37 | 0.0 (1.000) | 0.42 | 7.4 |  |  |  |
|  | MO | 40 | 0.0 (1.000) | 0.31 | 4.9 |  |  |  |
|  | PA | 40 | 0.0 (1.000) | 0.49 | 3.6 |  |  |  |
|  | WA | 39 | 0.0 (1.000) | 0.87 | 3.7 |  |  |  |
| Extra NO_2_ model 1 for 4 areas  (µg/m³, n=154) | BS | 40 | **3.1 (0.005)** | 0.51 | 4.7 | **4.0 (0.0002)** | 0.49 | 4.8 |
|  | GE | 38 | 0.0 (0.9842) | 0.41 | 9.0 | 0.1 (0.9471) | 0.41 | 9.0 |
|  | LU | 37 | -1.6 (0.1647) | 0.44 | 7.2 | **-2.8 (0.0151)** | 0.43 | 7.3 |
|  | WA | 39 | -1.7 (0.1270) | 0.81 | 4.5 | **-4.9 (<0.0001)** | 0.80 | 4.6 |
| PM_2.5_ absorbance model 1  (10^-5^ m^-1^, n=74) | BS | 20 | **0.08 (0.0469)** | 0.52 | 0.1 | **0.11 (0.0099)** | 0.48 | 0.10 |
|  | GE | 18 | **-0.09 (0.0447)** | 0.48 | 0.21 | **-0.13 (0.0063)** | 0.43 | 0.22 |
|  | LU | 17 | -0.01 (0.8692) | 0.38 | 0.22 | 0.02 (0.7251) | 0.27 | 0.24 |
|  | WA | 19 | 0.00 (0.9437) | 0.29 | 0.18 | 0.02 (0.7311) | 0.29 | 0.18 |
| PM_2.5_ absorbance model 2  (10^-5^ m^-1^, n=74) | BS | 20 | **0.18 (<0.0001)** | 0.41 | 0.11 | **0.27 (<0.0001)** | 0.40 | 0.11 |
|  | GE | 18 | -0.082 (0.0826) | 0.34 | 0.24 | -0.075 (0.1300) | 0.35 | 0.24 |
|  | LU | 17 | **-0.15 (0.0026)** | 0.60 | 0.18 | **-0.27 (<0.0001)** | 0.57 | 0.18 |
|  | WA | 19 | 0.018 (0.70) | 0.27 | 0.18 | 0.076 (0.1168) | 0.27 | 0.18 |
| PNC model 1  (particles/cm³, n=67) | BS | 17 | **2176 (0.0001)** | 0.34 | 854 | **3564 (<0.0001)** | 0.22 | 926 |
|  | GE | 16 | -856 (0.1108) | 0.70 | 2290 | **-1176 (0.0373)** | 0.68 | 2385 |
|  | LU | 16 | **-1791 (0.0012)** | 0.44 | 2492 | **-3167 (<0.0001)** | 0.42 | 2529 |
|  | WA | 18 | 298 (0.5522) | 0.48 | 1820 | **2236 (0.0001)** | 0.50 | 1778 |
| PNC model 2  (particles/cm³, n=67) | BS | 17 | **994 (0.0487)** | 0.36 | 836 | **1502 (0.0049)** | 0.35 | 847 |
|  | GE | 16 | -749 (0.1469) | 0.65 | 2471 | **-1276 (0.0191)** | 0.64 | 2523 |
|  | LU | 16 | -204 (0.6906) | 0.57 | 2190 | -447 (0.4032) | 0.57 | 2189 |
|  | WA | 18 | -92 (0.8482) | 0.38 | 1978 | **-1182 (0.0213)** | 0.39 | 1965 |
| LDSA model 1  (µm²/cm³, n=67) ^d^ | BS | 17 | **5.2 (<0.0001)** | 0.12 | 2.3 | **13.1 (<0.0001)** | 0.30 | 2.1 |
|  | GE | 16 | -0.1 (0.9561) | 0.65 | 3.7 | 0.8 (0.5448) | 0.61 | 3.9 |
|  | LU | 16 | **-6.8 (<0.0001)** | 0.53 | 4.6 | **-12.3 (<0.0001)** | 0.51 | 4.7 |
|  | WA | 18 | 1.1 (0.278) | 0.38 | 4.3 | **20.8 (<0.0001)** | 0.24 | 4.8 |
| LDSA model 2  (µm²/cm³, n=67) | BS | 17 | 0.61 (0.5255) | 0.10 | 2.3 | **1.2 (0.2219)** | 0.08 | 2.4 |
|  | GE | 16 | -0.29 (0.7719) | 0.58 | 4.0 | **-0.063 (0.9512)** | 0.57 | 4.1 |
|  | LU | 16 | -0.68 (0.4939) | 0.47 | 4.9 | **-1.0 (0.3265)** | 0.47 | 4.9 |
|  | WA | 18 | 0.28 (0.7631) | 0.44 | 4.1 | **1.5 (0.1257)** | 0.42 | 4.2 |

^a^ Predictions were made using the full model based on all available sites and applied to each area individually; ^b^ Predictions were made using the full model minus one area, while keeping the same predictors in the model, and letting the coefficients vary. This model was then applied to the left-out area.
