# Supplementary material for: Development of land use regression models for nitrogen dioxide, ultrafine particles, lung deposited surface area, and four other markers of particulate matter pollution in the Swiss SAPALDIA regions
Source: Environ Health. 2016 Apr 18;15:53. doi: 10.1186/s12940-016-0137-9 (PMC4835865; doi:10.1186/s12940-016-0137-9)
Supplement: Additional file 6: — Results of independent external validation for NO2, using different cut-off distances for the application of local versus alpine/non-alpine LUR models (n = 102). (DOCX 14 kb) [file 12940_2016_137_MOESM6_ESM.docx]

Additional file 6: Results of independent external validation for NO_2_, using different cut-off distances for the application of local versus alpine/non-alpine LUR models (n=102)

| Pollutant | Mean overprediction  (Standard deviation) | R² |
| --- | --- | --- |
| Predicted using the area-specific NO2 models if distance <20km to nearest site of that area, otherwise alpine (>1000m) or non-alpine (<1000m) models used ^a^ | -2.3 (6.4) | 0.72 |
| Predicted using the area-specific NO2 models if distance <10km to nearest site, of that area otherwise alpine (>1000m) or non-alpine (<1000m) models used ^b^ | -2.1 (5.8) | 0.76 |
| Predicted using the alpine (>1000m) or non-alpine (<1000m) models only ^c^ | -2.3 (6.0) | 0.72 |

^a^ NO_2_ LUR models were applied to 102 sites in total: the area-specific NO_2_ models were applied to 41 routine monitoring sites within 20km of the SAPALDIA measurement areas, alpine NO_2_ models were applied to 4 routine monitoring sites outside of SAPALDIA measurement areas, with altitudes above 1000m, and non-alpine NO_2_ models were applied to 57 routine monitoring sites outside of the SAPALDIA measurement areas, with altitudes below 1000m; ^b^ NO_2_ LUR models were applied to 102 sites in total: the area-specific NO_2_ models were applied to 26 routine monitoring sites within 10km of the SAPALDIA measurement areas, alpine NO_2_ models were applied to 4 routine monitoring sites outside of SAPALDIA measurement areas, with altitudes above 1000m, and non-alpine NO_2_ models were applied to 72 routine monitoring sites outside of the SAPALDIA measurement areas, with altitudes below 1000m; ^c^ NO_2_ LUR models were applied to 102 sites in total: alpine NO_2_ models were applied to 6 routine monitoring sites outside of SAPALDIA measurement areas, with altitudes above 1000m, and non-alpine NO_2_ models were applied to 96 routine monitoring sites outside of the SAPALDIA measurement areas, with altitudes below 1000m
