# Supplementary material for: Development of land use regression models for nitrogen dioxide, ultrafine particles, lung deposited surface area, and four other markers of particulate matter pollution in the Swiss SAPALDIA regions
Source: Environ Health. 2016 Apr 18;15:53. doi: 10.1186/s12940-016-0137-9 (PMC4835865; doi:10.1186/s12940-016-0137-9)
Supplement: Additional file 7: — Overview of model performance of LUR models built previously for Swiss study areas. (DOCX 60 kb) [file 12940_2016_137_MOESM7_ESM.docx]

Additional file 7: Overview and full discussion of model performance of LUR models previously built for Swiss study areas

## Model performance and comparison to other Swiss LUR models

For an overview summarizing the performance of all LUR models from the current study and from previous studies, see Additional File 7, Table 1. Prior to this study, multi-area and study area specific models were also developed for NO_2_ in all SAPALDIA areas following earlier measurement campaigns from the years 1993 and 2003.[1] In this previous study, model performance was reported only in terms of adjusted R², and we therefore report the R²_adj_ values of our study for comparison. This earlier study concluded that the multi-area NO_2_ models (1993: *R*^2^adj = 0.84; 2003: *R*^2^_adj_ = 0.83) did not explain equal amounts of spatial variability within the individual study areas (1993: *R*^2^_adj_ = 0.00 to 0.60; 2003: *R*^2^_adj_ = 0.17 to 0.73). In the current study, we found a lower *R*^2^_adj_ for the multi-area model (*R*^2^_adj_=0.51), yet this model was able to predict the spatial contrasts within the individual areas moderately well (*R*^2^=0.31-0.76) (Additional File 5). Despite that, the same model was unable to capture the between area contrasts adequately (Additional File 5), resulting in substantial prediction bias for some areas, as was also concluded in the earlier study [1]. This may be a result of long-range transport of pollutants, and could not be explained by the local predictor variables considered in our models. For the alpine and non-alpine NO_2_ models, the explained variance within each area was generally lower than the overall explained variance of the model (Additional File 5). This is likely a result of the increased range in concentration and predictor values on a national level as compared to the individual study areas, due to large between-area differences in topography, emissions, and meteorology. Yet, both the alpine and non-alpine models performed well in estimating the between-area contrasts (Additional File 3).

More recently, area-specific LUR models were developed for the ESCAPE project (2008-2010) for NO_2_ in Basel, Geneva and Lugano[2] and for PM_2.5_, PM_2.5_ absorbance, PM_10_ and PM_coarse_ in Lugano[3]. The local NO_2_ models developed for ESCAPE in Basel (R²=0.67), yielded a lower R² than in the present study (R²=0.78, Table 3).[2] This is probably due to a higher-quality national traffic model which was available for this study, whereas for ESCAPE, a node-network was used where roads were not geographically in the right locations, and traffic counts were not available for every road segment. Local ESCAPE models for Geneva (R²=0.87) and Lugano (R²=0.87) for which high quality traffic models were already available [2], yielded higher R²’s than observed for the present study (R²’s of 0.53 and 0.69, respectively, Table 3). Though we cannot explain the decreased model performance in Geneva and Lugano fully, a likely explanation for the generally lower performance of most SAPALDIA 3 models as compared to the ESCAPE models relates to the partly different strategies in the selection of measurement sites. While ESCAPE required all sampling to take place at the street side façade of the buildings, characterizing within-urban variability at the front door, SAPALDIA procedures targeted the outside environment where the SAPALDIA study subjects (once randomly selected from the population[4]) spent their time. In the SAPALDIA 3 campaign, PM and UFP measurements were taken in addition to NO_2_ at approximately 50% of sites in Basel, Geneva and Lugano. For the safety of equipment, these sites were located at balconies of the homes where SAPALDIA participants lived and may have taken place at the front, back or side of the building. However, no additional GIS-derived predictors were available to further characterize these micro-environments and possible shielding by buildings or trees. The ultimate use of the models is the application in health analyses of SAPALDIA or other health studies, and it is not known which exposure estimates are more relevant for health: those generated using a model based on the façade-points only, or those generated using a model based on where people are most likely to spend their time.

The same reason may explain why ESCAPE LUR models for Lugano also yielded higher R²’s for PM_2.5_ (R²=0.83), PM_10_ (R²=0.87) and PM_coarse_ (R²=0.77) [3]than were found in the current project (R² of 0.57, 0.63 and 0.45, respectively, Tables 3 and 4). For PM_2.5_ absorbance, the ESCAPE and current models performed similarly with R²’s of 0.79 and 0.81, respectively, but these statistics cannot be compared directly since we chose to fit multi-area models only. Similarly to the ESCAPE project[3] and several others[5], explained variance was higher for PM_2.5_ absorbance than for PM_2.5_ or PM_10_ mass. PM_2.5_ absorbance is a marker for black carbon which is majorly impacted by motorized road traffic in the direct vicinity, whereas PM_2.5_ and PM_10_ mass are more defined by longer-range transport. Lower model performance for PM_coarse_ can be attributed to the additional imprecision that is introduced by subtracting PM_2.5_ from PM_10_, and was previously noted in several other studies.[3, 6] In addition, it is possible that model performance is low because specific predictor variables related to sources of PM_coarse_ (abrasion of tires due to breaking and wear of different road surface materials) were not available.

Additional File 7, Table 1: Overview of model performance of LUR models previously built for Swiss study areas.

| LUR model | Study | N | R² | R²_adj_ ^a^ | LOOCV R² ^b^ | LOOCV RMSE ^b^ | Reference |
| --- | --- | --- | --- | --- | --- | --- | --- |
| Aarau NO_2_ | SAPALDIA 3 | 40 | 0.88 | 0.87 | 0.84 | 3.0 | This paper |
|  | SAPALDIA 2 2003 ^a^ | 31 | - | 0.69 | - | Not reported | [1] |
|  | SAPALDIA 1 1993 ^a^ | 60 | - | 0.60 | - | Not reported | [1] |
| Basel NO_2_ | SAPALDIA 3 | 40 | 0.78 | 0.76 | 0.64 | 4.0 | This paper |
|  | ESCAPE | 40 | 0.67 | - | 0.58 | 4.8 | [2] |
|  | SAPALDIA 2 2003 ^a^ | 37 | - | 0.47 | - | Not reported | [1] |
|  | SAPALDIA 1 1993 ^a^ | 85 | - | 0.61 | - | Not reported | [1] |
| Davos NO_2_ | SAPALDIA 3 | 38 | 0.73 | 0.69 | 0.62 | 6.9 | This paper |
|  | SAPALDIA 2 2003 ^a^ | 34 | - | 0.43 | - | Not reported | [1] |
|  | SAPALDIA 1 1993 ^a^ | 62 | - | 0.78 | - | Not reported | [1] |
| Geneva NO_2_ | SAPALDIA 3 | 38 | 0.53 | 0.49 | 0.43 | 8.9 | This paper |
|  | ESCAPE | 40 | 0.87 | - | 0.81 | 3.7 | [2] |
|  | SAPALDIA 2 2003 ^a^ | 39 | - | 0.52 | - | Not reported | [1] |
|  | SAPALDIA 1 1993 ^a^ | 36 | - | 0.55 | - | Not reported | [1] |
| Lugano NO_2_ | SAPALDIA 3 | 37 | 0.69 | 0.64 | 0.57 | 6.3 | This paper |
|  | ESCAPE | 42 | 0.87 | - | 0.82 | 3.5 | [2] |
|  | SAPALDIA 2 2003 ^a^ | 41 | - | 0.79 | - | Not reported | [1] |
|  | SAPALDIA 1 1993 ^a^ | 56 | - | 0.59 | - | Not reported | [1] |
| Montana NO_2_ | SAPALDIA 3 | 40 | 0.52 | 0.46 | 0.39 | 4.6 | This paper |
|  | SAPALDIA 2 2003 ^a^ | 40 | - | 0.78 | - | Not reported | [1] |
|  | SAPALDIA 1 1993 ^a^ | 42 | - | 0.22 | - | Not reported | [1] |
| Payerne NO_2_ | SAPALDIA 3 | 40 | 0.64 | 0.61 | 0.49 | 3.6 | This paper |
|  | SAPALDIA 2 2003 ^a^ | 45 | - | 0.89 | - | Not reported | [1] |
|  | SAPALDIA 1 1993 ^a^ | 51 | - | 0.73 | - | Not reported | [1] |
| Wald NO_2_ | SAPALDIA 3 | 39 | 0.89 | 0.89 | 0.86 | 3.9 | This paper |
|  | SAPALDIA 2 2003 ^a^ | 43 | - | 0.72 | - | Not reported | [1] |
|  | SAPALDIA 1 1993 ^a^ | 64 | - | 0.78 | - | Not reported | [1] |
| Multi-area NO_2_ | SAPALDIA 3 | 312 | 0.52 | 0.51 | 0.50 | 7.4 | This paper ^c^ |
|  | SAPALDIA 2 2003 ^a^ | 310 | - | 0.83 | - | Not reported | [1] |
|  | SAPALDIA 1 1993 ^a^ | 497 | - | 0.84 | - | Not reported | [1] |
| Lugano PM_2.5_ | ESCAPE | 19 | 0.83 | - | 0.77 | 1.1 | [3] |
| Multi-area PM_2.5_ | SAPALDIA 3 | 74 | 0.57 | 0.55 | 0.50 | 2.2 | This paper |
| Lugano PM_2.5_ abs | ESCAPE | 19 | 0.79 | - | 0.71 | 0.3 | [3] |
| Multi-area PM_2.5_ abs | SAPALDIA 3 | 74 | 0.81 | 0.79 | 0.77 | 0.19 | This paper |
| Lugano PM_10_ | ESCAPE | 18 | 0.87 | - | 0.80 | 1.6 | [3] |
| Multi-area PM_10_ | SAPALDIA 3 | 74 | 0.63 | 0.62 | 0.59 | 2.6 | This paper |
| Lugano PM_coarse_ | ESCAPE | 18 | 0.77 | - | 0.65 | 1.1 | [3] |
| Multi-area PM_coarse_ | SAPALDIA 3 | 74 | 0.45 | 0.43 | 0.38 | 1.6 | This paper |

^a^ Adjusted R^2^ was reported for in Liu et al. (2012), Dispersion + GIS model, overall and area-specific; ^b^ LOOCV R^2^ and RMSE’s from Liu et al. (2012) are not reported in the publication for the purely spatial (Dispersion + GIS) models, which are most comparable to the models presented in this paper and those of ESCAPE; ^c^ The statistics for the multi-area (eight-area) NO_2_ model from this paper refer to the first NO_2_ model reported in Additional File 5, Table 2, which considers, but does not force in NO_2_ dispersion model estimates.

References:

1. Liu S, Tsai M-Y, Keidel D, Gemperli A, Ineichen A, Hazenkamp-von Arx M, Bayer-Oglesby L, Rochat T, Künzli N, Ackermann-Liebrich U *et al*: **Long-term exposure models for traffic related NO2 across geographically diverse areas over separate years**. *Atmospheric Environment* 2012, **46**(0):460-471.

2. Beelen R, Hoek G, Vienneau D, Eeftens M, Dimakopoulou K, Pedeli X, Tsai M-Y, Künzli N, Schikowski T, Marcon A *et al*: **Development of NO2 and NOx land use regression models for estimating air pollution exposure in 36 study areas in Europe – The ESCAPE project**. *Atmospheric Environment* 2013, **72**(0):10-23.

3. Eeftens M, Beelen R, de Hoogh K, Bellander T, Cesaroni G, Cirach M, Declercq C, Dedele A, Dons E, de Nazelle A *et al*: **Development of Land Use Regression models for PM(2.5), PM(2.5) absorbance, PM(10) and PM(coarse) in 20 European study areas; results of the ESCAPE project**. *Environmental science & technology* 2012, **46**(20):11195-11205.

4. Martin B, Ackermann-Liebrich U, Leuenberger P, Künzli N, Stutz E, Keller R, Zellweger J-P, Wüthrich B, Monn C, Blaser K *et al*: **SAPALDIA: Methods and participation in the cross-sectional part of the Swiss Study on Air Pollution and Lung Diseases in Adults**. *Soz Präventivmed* 1997, **42**(2):67-84.

5. Hoek G, Beelen R, de Hoogh K, Vienneau D, Gulliver J, Fischer P, Briggs D: **A review of land-use regression models to assess spatial variation of outdoor air pollution**. *Atmospheric Environment* 2008, **42**(33):7561-7578.

6. Hoek G, Beelen R, Kos G, Dijkema M, Zee SCvd, Fischer PH, Brunekreef B: **Land use regression model for ultrafine particles in Amsterdam**. *Environmental science & technology* 2010, **45**(2):622-628.
